# Improving the activity and thermostability of PETase from *Ideonella sakaiensis* through modulating its post-translational glycan modification

**DOI:** 10.1038/s42003-023-04413-0

**Published:** 2023-01-13

**Authors:** Binyang Deng, Yu Yue, Jun Yang, Mingjun Yang, Qiong Xing, Hang Peng, Fei Wang, Ming Li, Lixin Ma, Chao Zhai

**Affiliations:** 1 State Key Laboratory of Biocatalysis and Enzyme Engineering, Hubei Collaborative Innovation Center for Green Transformation of Bio-resources, School of Life Sciences, Hubei University, Wuhan, People’s Republic of China; 2 School of Chemistry and Chemical Engineering, Hubei University, Wuhan, People’s Republic of China

**Keywords:** Biotechnology, Biochemistry

## Abstract

The large-scale preparation of Polyehylene terephthalate (PET) hydrolysing enzymes in low-cost is critical for the biodegradation of PET in industry. In the present study, we demonstrate that the post-translational glycosylation of *Pichia pastoris* makes it a remarkable host for the heterologous expression of PETase from *Ideonella sakaiensis* 201-F6 (*Is*PETase). Taking advantage of the abundant N- and O-linked glycosylation sites in *Is*PETase and the efficient post-translational modification in endoplasmic reticulum, *Is*PETase is heavily glycosylated during secretory expression with *P. pastoris*, which improves the specific activity and thermostability of the enzyme dramatically. Moreover, the specific activity of *Is*PETase increased further after the bulky N-linked polysaccharide chains were eliminated by Endo-β-N-acetylglucosaminidase H (Endo H). Importantly, the partially deglycosylated *Is*PETase still maintained high thermostability because of the remaining mono- and oligo-saccharide residues on the protein molecules. Consequently, the partially deglycosylated *Is*PETase was able to be applied at 50 °C and depolymerized raw, untreated PET flakes completely in 2 to 3 days. This platform was also applied for the preparation of a famous variant of *Is*PETase, Fast-PETase, and the same result was achieved. Partially deglycosylated Fast-PETase demonstrates elevated efficiency in degrading postconsumer-PET trays under 55 °C than 50 °C, the reported optimal temperature of Fast-PETase. The present study provides a strategy to modulate thermostable *Is*PETase through glycosylation engineering and paves the way for promoting PET biodegradation from laboratories to factories.

## Abstract (web summary)

Polyehylene terephthalate (PET) biodegradation is greatly improved when recombinant IsPETase is expressed in *Pichia pastoris* and the enzyme’s partial removal of N-terminal glycosylation, retaining high thermostability.

## Introduction

Polyehylene terephthalate (PET) is one the most used and synthesized plastics^1^. It is widely used in the textile, packaging, and bottle producing industries due to their high durability, elasticity, strength, and resistance to chemicals. On the other hand, the same characteristics caused serious problems in the natural degradation of PET^2^. Efficient, low-cost, and eco-friendly methods for the degradation of PET is in urgent need considering the increasing consumption and accommodation of PET in the environment. In 2005, Muller *et al* reported the biodegradation of PET with a PETase-like enzyme secreted by *Thermobifida fusca* for the first time^3^. Since then, many PET hydrolysing enzymes were identified and PET is by far the most studied polymer in terms of biodegradation^4^. Among these PET hydrolysing enzymes, PETase derived from *Ideonella sakaiensis* 201-F6, bacterium is able to use PET as the main carbon source^5^. However, this enzyme is thermosensitive and loses most of its activity within 24 h at 37 °C, which is unfavorable for the reaction since PET has a glass transition temperature (Tg) of 76 °C^6^. Based on the structure and the catalytic mechanism of *Is*PETase^7,8^, rational design and computational learning were carried out to improve its activity and thermostability. Among them, three outstanding variants were generated, namely ThermoPETase^9^, DuraPETase^10^ and Fast-PETase^11^. Especially the recent reported Fast-PETase, which bearing 5 mutations in comparison with wild-type *Is*PETase, is capable of depolymerizing untreated, postconsumer-PET from 51 different thermoformed products almost completely in 1 week. These remarkable achievements implied a bright prospect for the industrial biodegradation of PET in the near future. To transform the biodegradation of PET from laboratory to industry, large-scale preparation of enzymes under low-cost is crucial. The methylotrophic yeast *Pichia pastoris* (also known as *Komagataella phaffi*) is a commonly used workhorse in producing heterologous proteins for industries^12,13^. With the tightly regulated *AOX*1 promoter, efficient secretory expression, and the high-density fermentation technique, the yield of heterologous proteins can reach >20 g/L^14^. Moreover, the post-translational glycosylation of *P. pastoris* tends to increase the expression yield of the target proteins further because the glycoproteins are more resistant to proteinases, hence, facilitates the accumulation during the fermentation^15^. Plenty of examples have proved it is powerful in the large-scale preparation of biopharmaceuticals and industrial enzymes^14^.

In the present study, we prepared the recombinant *Is*PETase with *P. pastoris* GS115 as the host. Taking advantage of the gene dosage effect of the multicopy expression strategy, the yield of the target protein reached approximately 1.2 mg/mL with the high-density fermentation of 5-L fermentors. Moreover, we discovered the post-translational glycosylation of *P. pastoris* makes itself an excellent system for the preparation of *Is*PETase. The potential glycosylation sites in *Is*PETase were glycosylated during the secretory expression on varying degrees. Meanwhile, the high-level expression may prolong the retention time of the target protein in endoplasmic reticulum, leading to long polysaccharide chains. Therefore, the recombinant *Is*PETase (referred to as *Is*PETase-*Pp*) was heavily glycosylated, which promoted the folding and stability of *Is*PETase-*Pp*, leading to higher specific activity and thermostability than *Is*PETase expressed with *Escherichia coli* as the host (referred to as *Is*PETase-*Ec*). Moreover, the bulky N-linked glycan side chains of hyperglycosylation were removed easily with a glycohydrolase, Endo-β-N-acetylglucosaminidase H (Endo H, EC3.2.1.96) also prepared with *P. pastoris* in order to eliminate their blockage on the accessibility of the gigantic PET polymer to *Is*PETase. The specific activity of *Is*PETase-*Pp* enhanced further after the partial deglycosylation (referred to as partial-deglyco *Is*PETase-*Pp*) and increased more than 60-fold in comparison with *Is*PETase-*Ec*. More importantly, partial-deglyco *Is*PETase-*Pp* still maintained high thermostability because of the modulation of the remaining mono- and oligo-saccharide residues to the protein molecules. Due to the elevated specific activity and thermostability, the partially deglycosylated *Is*PETase-*Pp* was able to be applied on depolymerization of raw, untreated PET flakes completely in 2 to 3 days at 50 °C. Furthermore, this platform was also applied for the preparation of Fast-PETase and the same result was achieved. The recombinant Fast-PETase was heavily glycosylated and the partially deglycosylated Fast-PETase (referred to as partial-deglyco Fast-PETase-*Pp*) demonstrated elevated efficiency in depolymerizing postconsumer-PET trays at 55 °C, which was 5 °C higher than the optimal temperature of Fast-PETase expressed with *E. coli*^11^.

## Results

### The yield of the recombinant *Is*PETase increasing with the copy numbers of the target gene

The recombinant *P. pastoris* GS115 strains bearing 1 to 4 copies of *Is*PETase gene were constructed and named as GS115-IP-1, GS115-IP-2, GS115-IP-3, and GS115-IP-4, respectively. The target gene in these strains was expressed with shake-flask fermentation. The yield of the recombinant protein reached 0.13, 0.25, 0.48 and 0.60 mg/mL after 5-d induction, respectively. This result indicated that the expression level of the recombinant *Is*PETase increased with the gene dosage. SDS-PAGE of the fermentation supernatant indicated only smears were detected after 5-d induction (Supplementary Fig. 1a). Since it is the typical pattern caused by post-translational glycosylation of *P. pastoris*, we stained the gel with glycoprotein staining kit and the result proved our deduction (Supplementary Fig. 1b). The polysaccharide chains of *Is*PETase-*Pp* were removed with Endo H prepared in the present study (Supplementary Fig. 2). Obvious bands were observed after the treatment, which proved *Is*PETase-*Pp* was deglycosylated by Endo H successfully (Supplementary Fig. 1c). Glycoprotein staining also indicated the target protein was deglycosylated (Supplementary Fig. 1d). It is worth noting that the size of the deglycoprotein was slightly larger than the predicted molecular weight due to the remaining N-acetylglucosamine (GlcNAc) at N-linked glycosylation sites after Endo H treatment and O-linked glycans.

### The characteristics of partial-deglyco *Is*PETase-*Pp*

**Fig. 1:**
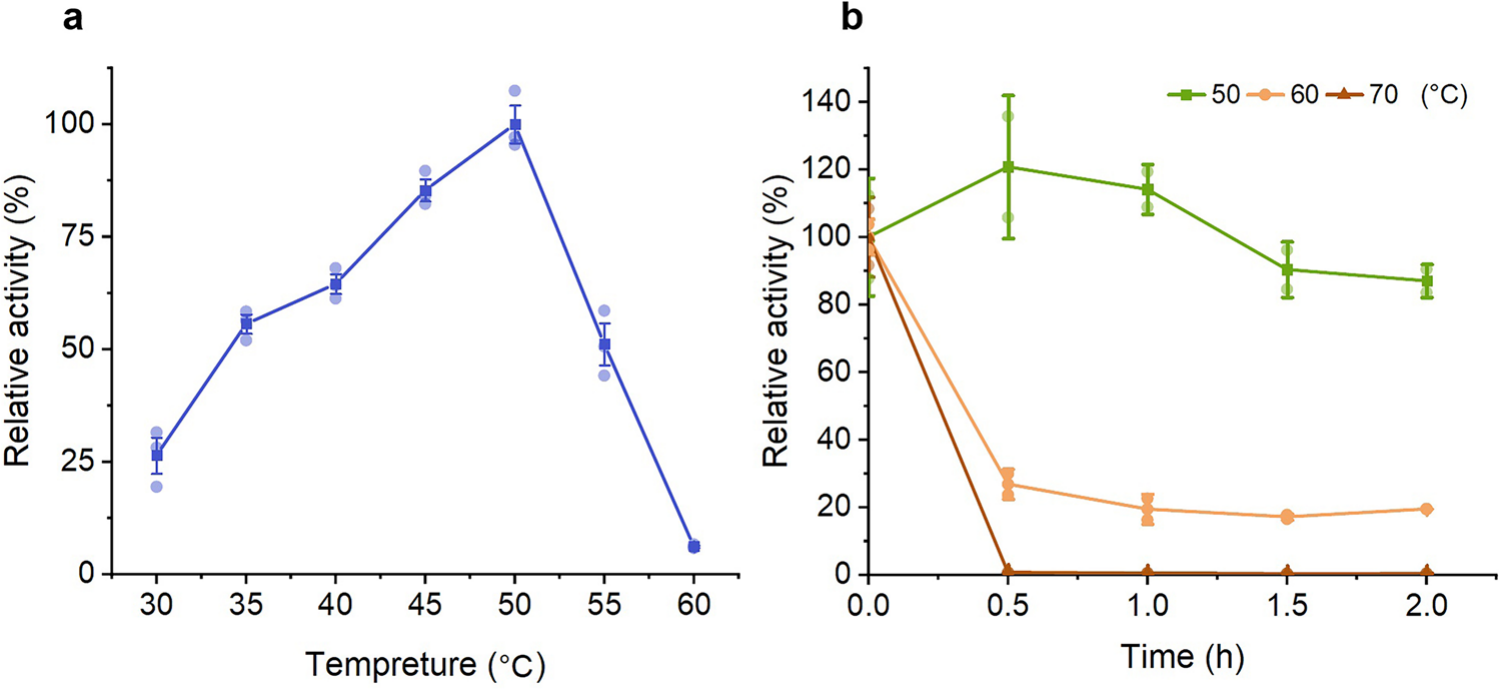
The optimum temperature of partial-deglyco *Is*PETase-*Pp* and the effect of the temperature on the stability of partial-deglyco *Is*PETase-*Pp*. **a** The optimum temperature of partial-deglyco *Is*PETase-*Pp*. The enzyme activity at 50 °C was set to 100%. **b** The effect of the temperature on the stability of partial-deglyco *Is*PETase-*Pp*. The activity of the enzyme without treatment was set to 100%. Data are presented as mean ± SD.

The optimum temperature of partial-deglyco *Is*PETase-*Pp* was 50 °C (Fig. 1a), which was higher than the previous report^5^. Moreover, partial-deglyco *Is*PETase-*Pp* displayed elevated thermostability in comparison with the previous report^16^. It remained approximately 90% of its activity after 2-h incubation at 50 °C. Its activity decreased dramatically at 60 and 70 °C, retaining less than 20% of the activity after 2 h (Fig. 1b).

### Preparation of *Is*PETase with the high-density fermentation

**Fig. 2:**
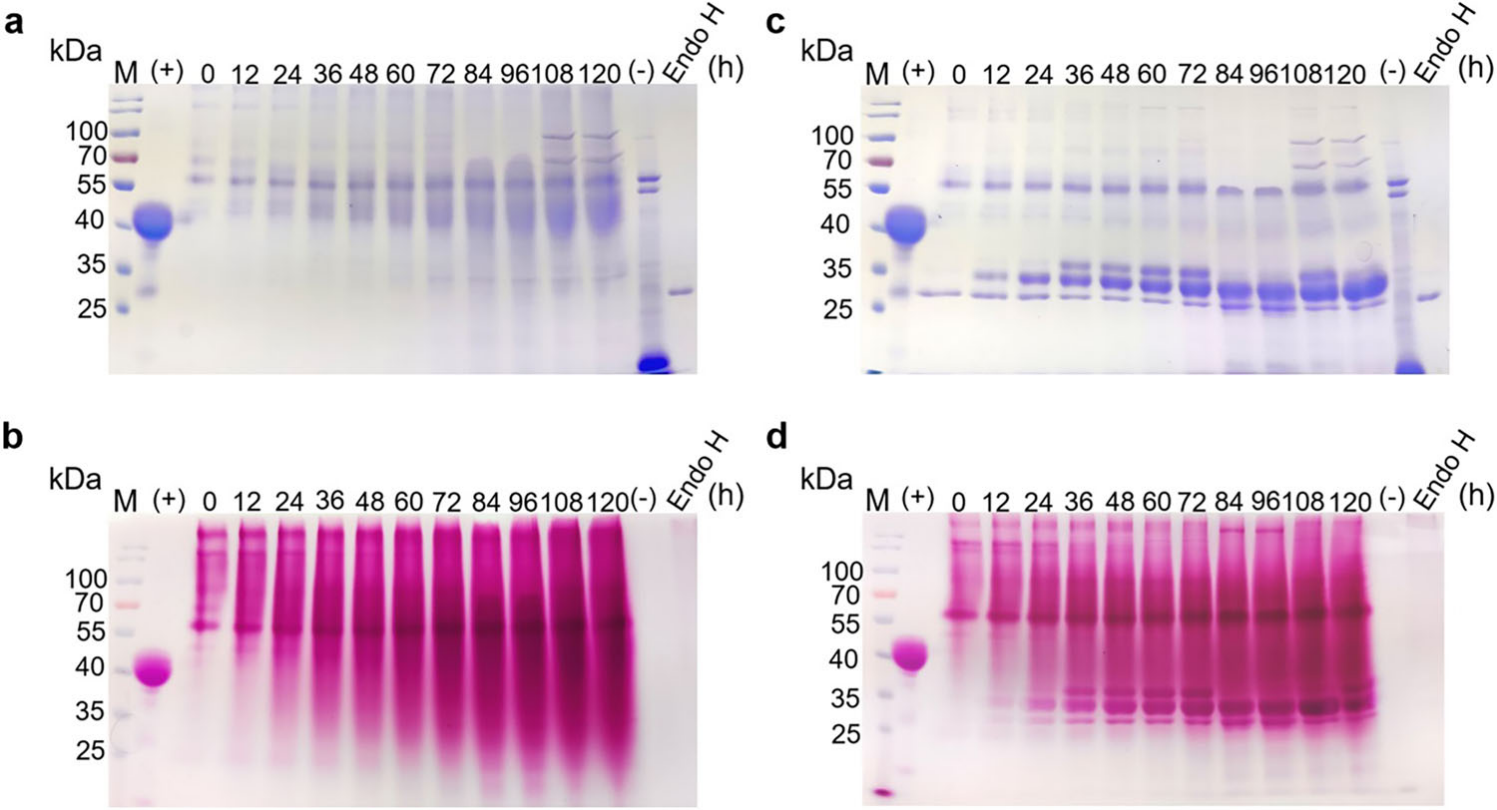
The expression and deglycosylation of *Is*PETase-*Pp* prepared with the high-density fermentation. **a** SDS-PAGE to analyze the expression of *Is*PETase-*Pp* during the high-density fermentation; **b** Glycoprotein staining to analyze the expression of *Is*PETase-*Pp* during the high-density fermentation. **c** SDS-PAGE to analyze the same samples from (**a**) after treated with Endo H. **d** Glycoprotein staining to analyze the same samples from (**a**) after treated with Endo H. M: protein molecular weight standards (the size of each band is indicated on the left). (+) the positive control (Horseradish Peroxidase); (-) the negative control (Soybean Trypsin Inhibitor).

The ingredients and cultivation conditions of the shake-flask fermentation is completely different from the large-scale fermentation in industry. Moreover, previous reports indicated high-level expression caused stress to the host cellular secretary pathway and the retaining time of the recombinant protein in the endoplasmic reticulum or the Golgi complex is extended, which in turn, causes the glycosyltransferase to elongate the polysaccharide chains and inhibit the catalytic activity of *Is*PETase-*Pp*. In order to investigate the glycosylation of *Is*PETase under industrial condition, *Is*PETase-*Pp* was prepared with high-density fermentation using GS115-IP-4. The expression level of *Is*PETase-*Pp* increased significantly compared with the shake-flask fermentation and reached maximum after 5 days, with a yield of approximately 1.20 g/L (Supplementary Fig. 3). Glycosylation staining indicated the target protein was heavily glycosylated (Fig. 2a, b). After the Endo H treatment, three main bands were observed (Fig. 2c, d). The result of MS indicated the two lower bands were *Is*PETase while the top band was a dihydrolipoyl dehydrogenase of *P. pastoris* (Supplementary Table 2).

### The catalytic activity and thermostability of partial-deglyco *Is*PETase-*Pp*

**Fig. 3:**
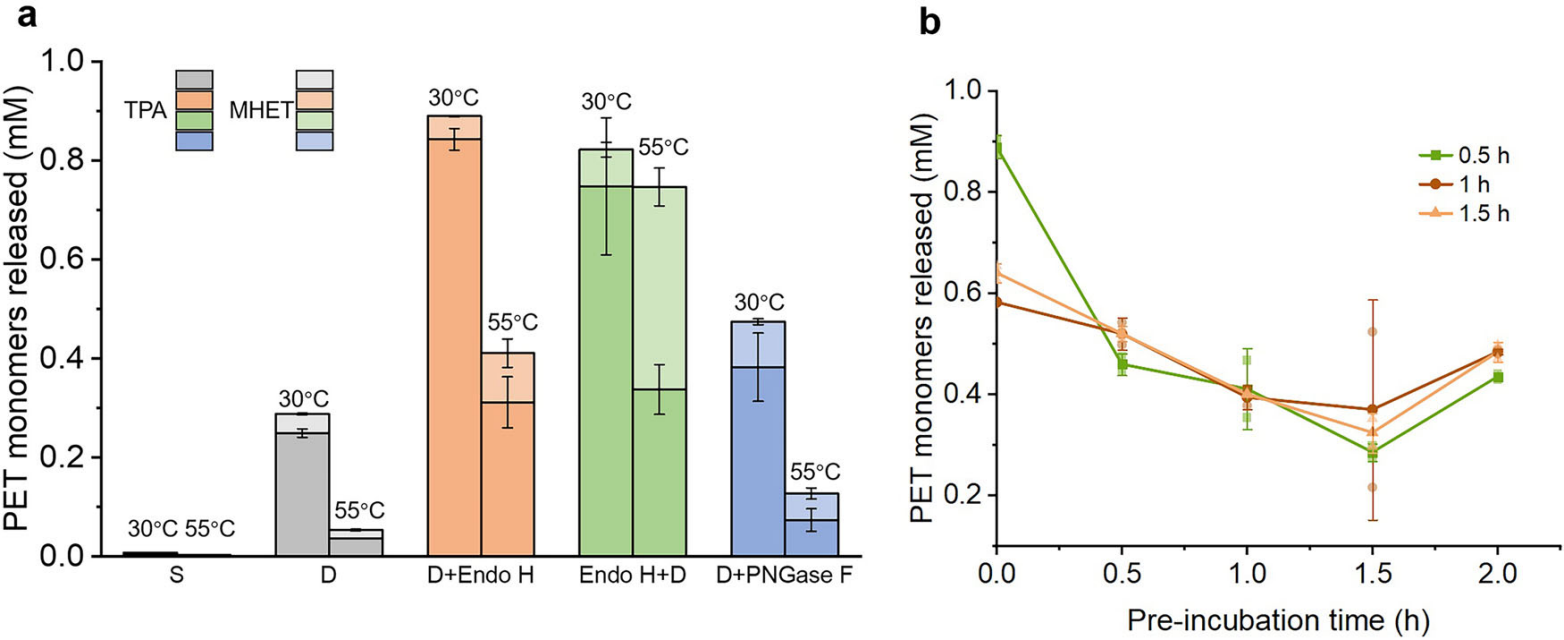
Optimization of the conditions for the deglycosylation of *Is*PETase-*Pp*. **a** The enzymatic activity of *Is*PETase-*Pp* with or without deglycosylation. S: the supernatant of the high-density fermentation; D: dialyzed supernatant of the high-density fermentation; D + Endo H: dialyzing prior to deglycosylation with Endo H; Endo H + D: deglycosylation with Endo H prior to dialyzing, D + PNGase: dialyzing prior to deglycosylation with PNGase F. 30 °C indicates the hydrolytic activity of the samples measured at 30 °C; 55 °C indicates the residual activity after incubated at 55 °C for 1 h. To analyze the enzyme activity, 0.4 mg of enzyme and 3 mg of amorphous PET were incubated in 1 mL of 50 mM glycine-NaOH buffer (pH 9.0) for 6 h at 30 °C. **b** The enzyme activity of *Is*PETase-*Pp* after treated with Endo H for 0.5, 1.0 and 1.5 h. The thermostability were measured after the samples were incubated at 55 °C for 0, 0.5, 1, 1.5, and 2 h. Data are presented as mean ± SD.

The catalytic activity of *Is*PETase-*Pp* with or without the Endo H treatment were investigated (Fig. 3a). The result indicated the fermentation supernatant barely had any activity because several metal ions in the cell culture, especially Fe^3+^ inhibited the catalytic activity of this enzyme dramatically (Supplementary Fig. 4). After ultrafiltration with a Millipore 10-kDa cut-off membrane, the activity of the enzyme increased obviously. Next, we treated the samples with Endo H and the activity increased dramatically. Notably, the activity of the sample treated with Endo H after ultrafiltration was slightly higher than the sample treated prior to ultrafiltration. But it was less thermostable after 1-h incubation at 55 °C. Therefore, either order is suitable for deglycosylation of *Is*PETase-*Pp*. Meanwhile, *Is*PETase-*Pp* was treated with another important glycohydrolase, PNGase F prepared in the present study (Supplementary Fig. 5). The result indicated the size of the product is smaller than partial-deglyco *Is*PETase-*Pp* using Endo H because PNGase F removes the whole N-linked glycans (Supplementary Fig. 6). The activity of *Is*PETase-*Pp* increased after the PNGase F treatment. But the activity and thermostability were lower in comparison with the Endo H treatment (Fig. 3a).

Meanwhile, we optimized the processing time of Endo H. *Is*PETase-*Pp* was treated with Endo H (final concentration of 667 nM) for 0.5, 1 and 1.5 h. The result indicated 0.5-h treatment demonstrated the highest initial activity (Fig. 3b). The thermostability of the samples was also investigated. Although the thermostability of the sample with 0.5-h treatment was weaker than the other two after incubated at 55 °C for 30 min, the residual activities at 2 h were similar, which implied 0.5-h treatment was enough. Taking all these results together, *Is*PETase-*Pp* after ultrafiltration was deglycosylated with 667 nM of Endo H for 0.5 h at 28 °C from now on. It is worth noting, Endo H only partially deglycosylated *Is*PETase-*Pp* under this condition and the product was a mixture of *Is*PETase bearing heterogeneous glycan side chains.

**Fig. 4:**
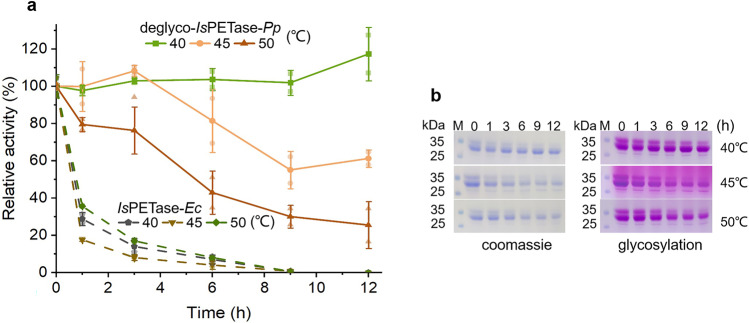
The thermostability of partial-deglyco *Is*PETase-*Pp*. **a** The effect of thermostability partial-deglyco *Is*PETase-*Pp* at 40, 45 and 50 °C. The residual activity was determined at 30 °C. The value at 0 min was taken to 100%. Measurements were repeated three times and averaged. **b** SDS-PAGE to analyze the degradation of partial-deglyco *Is*PETase-*Pp*. Data are presented as mean ± SD.

After treated with Endo H under the above-mentioned condition, the target protein was purified with Ni-NTA resin and its T_m_ value was identified with CD spectrum. T_m_ of partial-deglyco *Is*PETase-*Pp* was 62.05 °C (Supplementary Fig. 7), which is higher than *Is*PETase-*Ec* in the previous report^17^. Because the optimal temperature of partial-deglyco *Is*PETase-*Pp* was 50 °C, the thermostabilities of partial-deglyco *Is*PETase-*Pp* at 40, 45 and 50 °C were investigated. The result indicated that the enzyme was very stable at 40 °C and maintained full activity after 12 h. The protein remained approximately 60% of the activity after 12 h at 45 °C. At 50 °C, the enzyme retained more than 40% of its activity after 6 h (Fig. 4a). On the contrary, *Is*PETase-*Ec* lost almost full activity within 1 h under the same conditions. The result of SDS-PAGE was consistent with the data of the residual activities (Fig. 4b).

### The kinetics of partial-deglyco *Is*PETase-*Pp*

The specific activity of *Is*PETase-*Pp* increased from 20.6 U/mg to 62.4 U/mg after deglycosylation (Table 1), which was significantly higher than *Is*PETase-*Ec* (approximately 1.8 U/mg). This result indicated that post-translational glycosylation promoted the proper folding of the protein besides the thermostability. It is reported that the main hydrolytic products of *Is*PETase-*Ec* were TPA and MHET, which is consistent with our result. However, the major product (above 90%) of partial-deglyco *Is*PETase-*Pp* was TPA (Table 1). To investigate whether partial-deglyco *Is*PETase-*Pp* has elevated catalytic activity to converse MHET to TPA, the kinetic parameters of partial deglyco-*Is*PETase-*Pp* and *Is*PETase-*Ec* with MHET and BHET as the substrates were investigated. Although the catalytic turnover of partial-deglyco *Is*PETase-*Pp* was approximately 3.1-fold higher than *Is*PETase-*Ec*, the affinity of partial-deglyco *Is*PETase-*Pp* with BHET was approximately 2.7- fold lower, leading to a similar *k*_*cat*_/*K*_M_ for both enzymes. On the contrary, the catalytic turnover of partial-deglyco *Is*PETase-*Pp* was 2.4-fold higher than *Is*PETase-*Ec*, causing an approximately 3-fold increase in *k*_*cat*_*/K*_M_ for partial-deglyco *Is*PETase-*Pp* (Table 2, Supplementary Fig. 8). The higher *k*_*cat*_*/K*_M_ of partial-deglyco *Is*PETase-*Pp* explained why most of the hydrolytic product was TPA.

**Table 1:** The specific activity of the recombinant *Is*PETase.

Enzyme	TPA (mM)	MHET (mM)	Percentage of TPA (%)	specific activity (U/mg)
*Is*PETase-*Ec*	0.0023 ± 0.0004	0.0039 ± 0.0004	36.84 ± 1.72	1.0 ± 0.17
*Is*PETase-*Pp*	0.2492 ± 0.0061	0.0386 ± 0.0011	86.60 ± 0.06	20.6 ± 0.74
partial-deglyco *Is*PETase-*Pp*	0.8427 ± 0.0154	0.0469 ± 0.0012	94.72 ± 0.02	62.4 ± 1.64

The results are presented as the means ± SDs of three independent experiments. To analyze the enzyme activity, 0.4 mg of enzyme and 3 mg of amorphous PET were incubated in 1 mL of 50 mM glycine-NaOH buffer (pH 9.0) at 30 °C for 6 h.

**Table 2:** *Is*PETase kinetics for MHET and BHET.

	MHET			BHET		
Enzyme	*K*_M_	*k*_*cat*_	*k*_*cat*_/*K*_M_	*K*_M_	*k*_*cat*_	*k*_*cat*_/*K*_M_
partial-deglyco *Is*PETase-*Pp*	0.84 ± 0.09	37.99 ± 4.14	45.23	0.88 ± 0.17	40.14 ± 7.55	45.61
*Is*PETase-*Ec*	1.04 ± 0.07	15.61 ± 1.03	15.01	0.30 ± 0.09	12.81 ± 3.81	42.70

The results are presented as the means ± SDs of three independent experiments.

### Analysis of the glycosylation modification in *Is*PETase-*Pp*

There are 8 potential N-linked glycosylation sites in *Is*PETase (Supplementary Fig. 9). These sites scatter on the surface of *Is*PETase and locate on the coils linking β-sheets and α-helixes. These features facilitate the N-glycosylation of the proteins and explain the heavy glycosylation of *Is*PETase. The glycan modification in *Is*PETase-*Pp* was investigated with MS/MS. The N-linked modification sites and main types of the polysaccharide chains are listed in Supplementary Table 3 and 4. The result indicated that N-linked glycosylation was detected with all eight potential sites. Five out of the eight potential glycosylation sites, N114, N138, N212, N264 and N288 showed high signal while the signals of N190, N205 and N277 were much weaker (N stands for Asn), which was detected when N-linked glycan score was set as 0 (Supplementary Table 3A and 4A). The degree of glycosylation was different with each site (Supplementary Table 3B and 4B). Notably, Asp206 next to Asn205 belongs to the catalytic triad of *Is*PETase. Since the signal of N205 was weak (Supplementary Table 4C, Supplementary Fig. 10), we proposed the effect of glycosylation to the hydrolytic center was slight, which implied the glycosylation inhibited the catalytic activity of the enzyme through shielding the enzyme molecules from the substrate rather than blocking the catalytic site. Meanwhile, the result also demonstrated that there are 19 O-linked glycosylation sites on *Is*PETase-*Pp* (Supplementary Table 5). For O-linked glycosylation, oligosaccharides are attached to Ser or Thr residues through glycosidic linkages. Therefore, the pattern was more complex. However, O-linked glycans were relatively shorter (Supplementary Table 5C) and the ratio of glycosylated protein were lower in comparison with N-linkage (Supplementary Table 5B), hence the shielding effect was much weaker, and explained why Endo H treatment was enough to recover the activity of the protein.

Based on the result of MS/MS, we constructed the three-dimensional structure of the glycoprotein at GLYCAM-Web (Supplementary Fig. 11). Although the protein is covered with polysaccharide chains, fortunately the facet located the triad and the key residues involved in catalysis had much less modification than the other facets.

### Expression of Fast-PETase with *P. pastoris* GS115

**Fig. 5:**
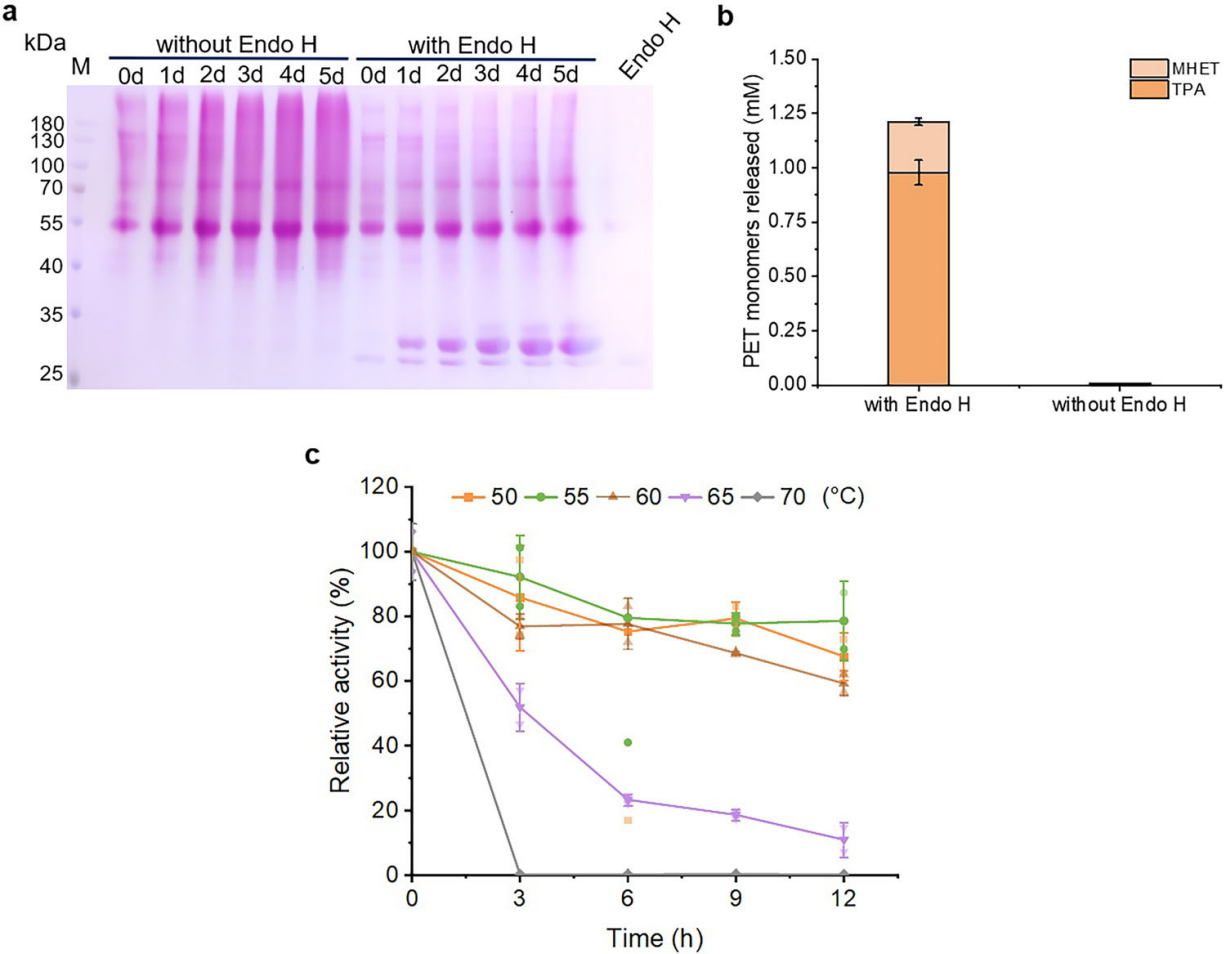
Preparation of Fast-PETase-*Pp* with the high-density fermentation. **a** Glycoprotein staining to analyze the expression of Fast-PETase during the high-density fermentation. The supernatant of the cell culture was collected every 24 h and treated with Endo H for half an hour at 30 °C. **b** The activity of Fast-PETase-*Pp* before and after Endo H treatment. **c** The thermostability of partial-deglyco Fast-PETase-*Pp*. The activity of the enzyme without treatment was set to 100%. Data are presented as mean ± SD.

Recently, an *Is*PETase variant, Fast-PETase with much higher thermostability and activity was generated^11^. We chose this variant to test the generalizability of this PETase preparation strategy. There is no overlapping between the glycosylation sites (including O- and N- linked) and the mutation sites in Fast-PETase (Supplementary Fig. 12). The result of SDS-PAGE indicated that Fast-PETase-*Pp* was also heavily glycosylated with either the shake-flask (Supplementary Fig. 13) or high-density fermentation (Fig. 5a). With the high-density fermentation, the yield of the target protein reached 0.4 mg/mL using single copy of the Fast-PETase gene. The variant barely had any activity even after the ultrafilitration and its activity was elevated significantly after Endo H treatment (Fig. 5b). In addition, partial-deglyco Fast-PETase-*Pp* demonstrated high thermostability. It retained above 80%, 70% and 60% of the catalytic activity after incubated at 50, 55 and 60 °C for 12 h, respectively. Its thermostability decreased dramatically at 65 °C and retained only 10% of activity after 12 h. At 70 °C, the full activity was lost within 3 h (Fig. 5c).

### Depolymerization of the raw, untreated postconsumer-PET

**Fig. 6:**
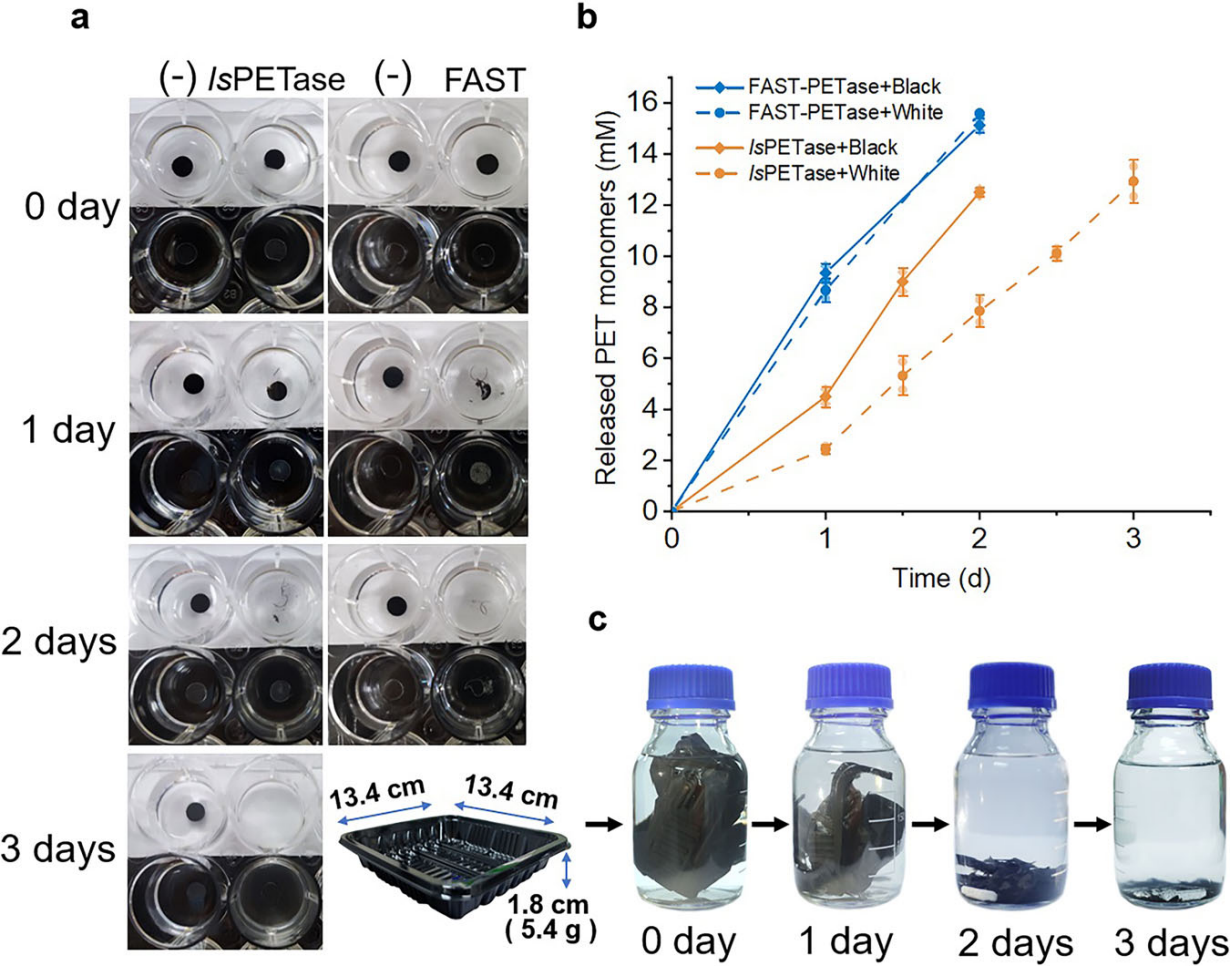
Depolymerization of the raw, untreated postconsumer-PET. **a** Degradation of raw, untreated PET flakes with *Is*PETase-*Pp* and partial-deglyco FAST-PETase-*Pp* at 50 °C. **b** Time course of PET monomers released from the flakes during the digestion with partial-deglyco *Is*PETase-*Pp* and partial-deglyco FAST-PETase-*Pp*. **c** Degradation of an untreated PET tray with partial-deglyco FAST-PETase-*Pp* at 50 °C.

The ability to depolymerize postconsumer-PET, such as containers and bottles is the primary need in practice. Therefore, the recombinant *Is*PETase and Fast-PETase were applied to degrade commercial raw, untreated postconsumer-PET. The PET trays were purchased online randomly. The percentage crystallinity of the black and white trays are 13.34% and 9.04%, respectively (Supplementary Fig. 14). The experiments were carried out at 50 °C. Partial-deglyco *Is*PETase-*Pp* retained approximately 40% and 20% activity after 6 and 12 h at 50 °C. Therefore, fresh enzyme was added every 12 h. Partial-deglyco *Is*PETase-*Pp* depolymerized a hole-punched black flake in 2 days and a white flake in 3 days (Fig. 6a). The result of HPLC indicated PET was degraded in an almost linear speed and the major product was TPA (Fig. 6b). Just as its name stated, partial-deglyco Fast-PETase-*Pp* degraded raw PET even faster. In 2 days, a white flake or a black flake was completely degraded with replenishing enzyme once. In addition, a black tray of 5.4 g was depolymerized in 3 days with partial-deglyco Fast-PETase-*Pp* at 50 °C (Fig. 6c). Moreover, partial-deglyco Fast-PETase-*Pp* demonstrated even higher efficiency at 55 °C in comparison with 50 °C (Supplementary Fig. 15), which is consistent with the result of the thermostability assay. Neither enzyme displayed visible degradation to commercial PET water bottles in 2 days.

## Discussion

The glycosylation of proteins is common in yeasts. In this post-translational modification process, proteins are passed into the endoplasmic reticulum cotranslationally and glycosylated, followed by sending to the Golgi complex for further processing, and then are either targeted to various organelles, become plasma membrane components, or secreted into the periplasm^15^. Previous reports indicated the covalent binding of glycans to the protein surface increase solubility of the protein and its resistance to proteolysis. The glycans also enhance the thermodynamic and kinetic stability of the target protein^18^. However, quantification and generalization of these effects and the molecular components that dictate the capability of the attachments to modulate protein characteristics are still lacking^19^. In *P. pastoris*, majority of the oligosaccharide modification belong to N-linked glycans and only a small portion is O-glycosidic linkage in most of the glycoproteins^20^. To date, the O-linked glycans in this organism is still ambiguous^21^. On the other hand, more information was gained about the N-linked glycans. In most cases, N-linked glycosylation happens on an Asn residue as part of the consensus sequence Asn-X-Ser/Thr (where X is any amino acid except Pro). Previous reports also indicated that occupied glycosylation sites tend to be located in flexible regions of the protein. Moreover, it is hypothesized that the greater stabilization effect is achieved when the attached glycan is located at a more flexible region (e.g. a loop) of the protein while a significant destabilization occurs if the glycosylation site is located at a highly structured region^22^. Seven out eight of the N-linked glycan sites in *Is*PETase are located on the loops of the protein surface. These theories explained why *Is*PETase was heavily glycosylated and *Is*PETase-*Pp* displayed significantly higher specific activity and thermostability in comparison with *Is*PETase-*Ec*. The glycosylation of another famous PET hydrolyase, LCC (Leaf and branch compost cutinase) also led to improved stability to native state aggregation and increased the temperature for thermal induced aggregation by 10 °C when expressed with *P. pastoris*^23^. The enhanced activity of deglycosylated BurPL-DM and *Is*PETase expressed using *P. pastoris* has been reported previously^24^, but the increase is not as obvious as the present study. It is likely the ingredient and condition of the high-density fermentation facilitated the glycosylation of *Is*PETase and improved its characteristics more significantly.

The reasons why the partially deglycosylated *Is*PETase-*Pp* has higher hydrolytic activity than *Is*PETase-*Pp* are complex. The N-linked glycan on Asn205 is likely to affect the enzymatic activity because Asp206 residue belongs to the triad of *Is*PETase. However, Asn205 happened to be occupied at very low level. The reason is unknown. But it implied it is not the main reason for the inhibition. On the other hand, the bulky N-linked glycans could block the interaction between the substrate with the enzymes. Consistent with this hypothesis, *Is*PETase-*Pp* couldn’t be purified with Ni-NTA affinity chromatography while partial-deglyco *Is*PETase-*Pp* was capable to be purified under the same condition. Therefore, we don’t suggest to improve the catalytic activity by simply eliminating the glycan side chains attached to Asn205 through site-directed mutagenesis. Previously, we developed a method to remove N-linked polysaccharide side chains from the enzymes in the supernatant of the high-density fermentation with Endo H directly^17^. Because commercial Endo H is very expensive and unsuitable for the large-scale deglycosylation of industrial enzymes, we used *P. pastoris* to prepare Endo H. Without purification, this recombinant endoglycosidase is able to remove N-linked polysaccharide side chains from glycoproteins in the supernatant of the high-density fermentation of *P. pastoris*. In the present study, we used this strategy to enhance the activity of the recombinant *Is*PETase. *Is*PETase-*Pp* still retained high thermostability after the long polysaccharide chains were removed. There is a GlcNAc residue left after the Endo H cleavage. The remaining monosacharides are the key. Previous reports indicated that the first two to three saccharides of large glycans often contribute almost all the stabilization effect^25^. Our previous study with a chitosanase demonstrated glycosylation with a single N-linked site increased the thermostability of the enzyme significantly^26^. Therefore, it is rational to found increasing thermostability with the partially deglycosylated *Is*PETase-*Pp* bearing short oligosacharides. We tried to remove the polysaccharide side chains with PNGase F, an amidase which cleaves between the innermost GlcNAc and Asn residues and moves almost all N-linked oligosaccharides from glycoproteins. The result indicated the polysaccharide side chains were cut off efficiently while the activity and thermostability of the deglycoprotein were decreased in comparison with the Endo H treatment. The previous report indicated that mutating the glycosylation sites of a protein, ribophorin I in vivo reduced the efficiency of folding and increased degradation^27^. Therefore, we proposed that both post-translational glycosylation of *P. pastoris* and partial deglycosylation with Endo H are equally important in the preparation of the recombinant *Is*PETase. This is also another reason why we didn’t mutate Asn205.

Another main concern with the large-scale preparation of industrial enzymes is the yield of the target protein. Previously, *Is*PETase was expressed in *E. coil* with a novel fusion tag, carbohydrate-binding module 66 (CBM66) to improve the soluble expression, and the titer of the recombinant *Is*PETase reached 370 mg/L^28^. *Is*PETase was also expressed with microalgaes as host, such as *P. tricornutum* and *C. reinhardtii* and the recombinant protein can be detected with western blot^29^. Meanwhile, secretory expression of *Is*PETase with *B. subtilis* or *E. coli* as hosts were performed^12,30–32^ and the target protein were detected with SDS-PAGE after highly concentrated. In the present study, we constructed expression vectors bearing 1-4 tandem expression cassettes to improve the yield of *Is*PETase with gene dosage effect. The preliminary result displayed a trend that the expression of *Is*PETase increased with the copies of the target gene. Increasing the yield of the target protein through multicopy replicons is an efficient strategy commonly used in *P. pastoris* heterologous expression^33^. Further study about the coordination of the gene copies and mRNA/protein level is in need in order to maximize the gene dosage effect. In addition, the condition of the high-density fermentation will be optimized further to improve the yield of *Is*PETase in the future.

In summary, we provided a strategy for the large-scale preparation and modulation of *Is*PETase and its variants. This work paved the way for promoting biodegradation of PET from laboratories to factories.

## Methods

### Strains, plasmids, media and reagents

*E. coli* DH5α and XL10-gold for gene cloning were stored in our lab. *E. coli* BL21 (DE3) and the expression vector pET28a were from Novagen (USA). Luria–Bertani (LB) medium for the cultivation of *E. coli* was prepared as described in the Manual of Molecular Cloning^34^. Expression vectors pHBM905BDM and pHBM905BDM-EndoH are stored in our lab. *P. pastoris* GS115 were obtained from Invitrogen (USA). The recombinant *P. pastoris* GS115 for the expression of Endo H was stored in our lab. Buffered glycerol-complex medium (BMGY), Buffered methanol-complex medium (BMMY) and minimal dextrose medium (MD) for the cultivation of yeast were prepared according to the *Pichia* Expression Kit (Invitrogen, USA). Terephthalic acid (TPA) was purchased from Sinopharm (China) and Monohydroxyethyl terephthalate (MHET) was purchased from Macklin (Shanghai, China). Amorphous PET (CAS25038-59-9) was purchased from Cool Chemistry (China) and granulated to smaller size (13-23 microns) with a blender. All other reagents used in this study were analytical reagents.

### Expression and purification of *Is*PSTase and PNGase F with *E. coli* as the host

The coding sequence of *Is*PETase without the signal peptide (Supplementary Table 1) was optimized based on the codon usage preference of *E. coli* and synthesized, followed by cloning into pET21b vector by Qinke (China). A CL8 tag^35^ fused to its N-termini to improve its solubility and a 6×His tag fused to its C-termini to facilitate the purification (Supplementary Table 1, pET21b-CL8-IsPETase, Addgene ID 195565). The coding sequence of PNGase F (Supplementary Table 1) was cloned into pET28a (pET28a-CL7-PNGase F, Addgene ID 195563). A CL7 tag^35^ fused to its N-termini to improve its solubility and a 6×His tag fused to its C-termini for affinity purification.

The recombinant plasmids were verified by Sanger sequencing, followed by transforming into *E. coli* BL21(DE3). To induce the expression of the target genes, the transformants were incubated at 37 °C to OD_600_ of 0.6-0.8, and then induced with 1 mM of Isopropyl-β-D-thiogalactopyranoside (IPTG) at 18 °C for 24 h. Cells were collected and resuspended in lysis buffer (50 mM Tris-HCl; 150 mM NaCl; 1 mM PMSF, pH 7.5) with lysozyme to a final concentration of 1 mg/mL. Samples were ultrasonicated to break the cells, followed by incubating with HRV 3 C at 18 °C overnight to remove the CL8 tag. Next, the samples were centrifugated at 13,000 rpm for 10 min and the supernatant was applied to Ni-NTA beads for affinity purification. The column was washed twice with 2 column volume of wash buffer (50 mM Tris-HCl; 150 mM NaCl; 20 mM imidazole, pH 7.5). One column volume of elution buffer (50 mM Tris-HCl; 150 mM NaCl; 100 mM imidazole, pH 7.5) was used to recover the target protein. The sample was then collected and dialyzed with a Millipore 10-kDa cut-off membrane at 4 °C to remove ions and salts, followed by resuspending with storage buffer (50 mM Tris-HCl; 150 mM NaCl, pH 7.5).

### The construction of yeast expression vectors and the transformation of *P. pastoris* GS115

*Is*PETase coding sequence including the signal peptide region was optimized based on the usage preference of *P. pastoris* and cloned into pHBM905BDM vector for secretory expression (Supplementary Table 1, pHBM905BDM-*Is*PETase, Addgene ID 195561). The multicopy expression vectors bearing 2, 3, and 4 copies of the *Is*PETase expression cassettes were constructed with the bio-brick method as previously reported^36^. To express Fast-PETase (Supplementary Table 1), the ORF of Fast-PETase generated through overlapping PCR with *Is*PETase ORF as the template, followed by cloning into pHBM905BDM (pHBM905BDM-Fast-*Is*PETase, Addgene ID 195561). To generate the recombinant *P. pastoris* strains, the recombinant plasmids were linearized with *Sal*I and transformed into *P. pastoris* GS115 by electroporation (7000 V/cm, 25 mF, 400 Ω; Life technologies cell porator, USA). Transformants were selected on MD plates (without histidine). The recombinants were identified with whole-cell PCR.

### The shake-flask fermentation

The Transformants were incubated for 2 days at 28 °C in 50 mL of BMGY medium before pelleting the cells by centrifugation (4000 × g, 5 min) and replenishing with 50 mL of BMMY. To induce the expression of the target protein, methanol was added to a final concentration of 1% (v/v) every 24 h. The supernatant was harvested after 5 days of induction by centrifugation at 4 °C and 10000 × g for 5 min.

### The high-density fermentation

Fed-batch fermentation was performed according to the Invitrogen *Pichia* fermentation process guidelines (USA). The recombinant *P. pastoris* strain was inoculated into individual flask containing 200 mL of BMGY, and the culture was incubated at 200 rpm, 28 °C for 24 h. The cell culture was then transferred into 5-L fermentors containing 3 L of BSM medium (BG-4, Baoxing, China). During the early stage of the fermentation, the culture was maintained at 28 °C, pH 5.8, with 30% dissolved oxygen (DO). After approximately 18 to 24 h, the glycerol was exhausted, and the DO was increased to 100% rapidly. To continue the cell growth, 50% (v/v) glycerol supplemented with PTM1 trace salts (12 mL/L) was added to the cultures at a speed of 12 mL/h/L, and the DO was kept above 20%. When the wet weight of the cells increased to approximately 180 g/L, the expression of the target gene was induced with methanol. The fermentation conditions were adjusted to 25 °C at pH 5.5, and the DO was set to 20–30%. Meanwhile, methanol with PTM1 trace salts (12 mL/L) was fed to the cells at a rate of 3 mL/h/L. After 2 h, the rate was increased by 20% per hour until it reached 7 mL/h/L. This condition was maintained until the end of the fermentation.

### Deglycosylation of the recombinant proteins

Deglycosylation of glycoproteins was carried out as previously described with modification^17^. Briefly, the supernatant of the cell culture was collected after 5-day induction with methanol and dialyzed using a Millipore 10-kDa cut-off membrane device. Next, the protein concentration in the supernatant was determined using the Bradford kit (Beyotime, China). Bovine serum albumin was used as the standard. And then, the recombinant Endo H was added at a final concentration of 667 nM. The mixture was incubated at 28 °C for half an hour. The digestion of PNGase F was carried out at a final concentration of 2.67 μM and incubated at 37 °C for 2 h.

### SDS-PAGE and glycoprotein staining

The samples were separated via sodium dodecyl sulfate-polyacrylamide gel electrophoresis (SDS-PAGE) using 12% (w/v) polyacrylamide gels, followed by staining with Coomassie Brilliant Blue G-250. Glycoprotein staining was performed as described in the Glycoprotein Staining Kit (Beyotime, China). Equal amount of each sample was loaded onto two 12% (w/v) poly-acrylamide gels. Following SDS-PAGE, the gels were stained with Coomassie brilliant blue G-250 and the Glycoprotein Staining Kit, respectively.

### Mass spectrum (MS)

Samples were loaded on a 12% (v/v) polyacrylamide gel for SDS-PAGE, followed by staining with Coomassie brilliant blue G-250. The gel slice including the target band was cut out and digested with trypsin overnight at 37 °C, followed by analyzing with Q Exactive^TM^ HF mass spectrometry. The electrospray voltage was 2.0 kV. The m/z scan range was 350 to 1800 for full scan, and intact peptides were detected in the Orbitrap at a resolution of 60,000. The result of MS was processed with Proteome Discoverer 2.4 software, and searched against *Komagataella phaffi* database.

### Analysis of the glycan side chains of *Is*PETase-*Pp* and structural modeling of the glycoprotein

The supernatant from the high-density fermentation was collected and dialyzed with a Millipore 10-kDa cut-off membrane at 4 °C to remove ions and salts, followed by resuspending with 50 mM Tris-HCl (pH 7.5). The sample was sent to Genecreate (China) for the Glycosylation analysis. The sample was incubated with DTT (10 mM) at 74 °C for 30 min, followed by adding 20 mM of IAA to block the free sulfhydryl groups of cysteine residues. Extra IAA was neutralized with DTT, followed by digestion with multiple enzymes. After the treatment, the product was centrifuged at 1,2000 rpm for 2 min and the supernatant was lyophilized. The sample was separated with HPLC and analyzed with tims TOF Pro2 mass spectrum. The data were collected with CID method and analyzed with PEAKS GlycanFinder software.

The three-dimensional structure of the glycosylated *Is*PETase was constructed at GLYCAM-Web (www.glycam.org) and the figure was illustrated with UCSF chimera software.

### Far-ultraviolet circle dichroism (CD)

Far-ultraviolet CD was performed with Chirascan V100 circle dichroism spectrometer (Applied Photophysics, UK). The protein was purified and adjusted to a concentration of 0.25 mg/mL with Tris-NaCl buffer (pH 7.5). The sample was scanned within wavelength of 190~245 nm from 20 to 100 °C with an interval of 10 °C. The thermal melting curve was monitored at 222 nm from 20 to 80 °C with an interval of 5 °C. The melting temperature was estimated using simple two-state model of OriginPro 8.0 software (OriginLab Corporation USA).

### Analysis of PET-hydrolytic activity using granular PET

The enzyme activity of *Is*PETase was measured as previously described^7^. Briefly, 200 µL of the diluted enzyme sample was mixed with 3 mg of amorphous PET (percentage crystallinity of 25.7%) in 50 mM glycine-NaOH buffer (pH 9.0) to a final volume of 1 mL, followed by incubating at 30 °C for 6 h. The reaction was terminated by heating at 85 °C for 10 min, and the mixture was centrifuged at 14000 g for 1 min, followed by analysis with HPLC. The activity of Fast-PETase was analyzed with the same method except the buffer was changed to 100 mM KH_2_PO_4_ buffer (pH 8.0). TPA and MHET released from the PET depolymerization was quantified with HPLC. A Prominence Ultra Fast Liquid Chromatograph (Shimadzu) equipped with an GL Sciences InertSustain C18 column (4.6 × 250 mm, 5 µm). The mobile phase was methanol with 20 mM phosphate buffer (pH 2.5) at a flow rate of 0.8 mL/min. The elution condition was 0–25 min with 25–85% (v/v) methanol linear gradient. The effluent was monitored at a wavelength of 240 nm. A standard curve was plot using TPA and MHET. All of the experiments were carried out in triplicate. One unit of enzyme (U) was defined as the amount of enzyme causing the release of 1 µg of TPA and MHET at 30 °C for 1 h.

### Analysis of the thermostability of *Is*PETase and Fast-PETase

To analyze the thermostability of *Is*PETase from the shake-flask fermentation, the supernatant was collected after 120 h and dialyzed with a Millipore 10-kDa cut-off membrane at 4 °C and the protein concentration in the protein was determined using the Bradford kit (Beyotime, China), followed by digestion with Endo H for half an hour. Next, 0.1 mg of the enzyme was incubated in 1 mL of 50 mM glycine-NaOH buffer (pH 9.0) at 50, 60 and 70 °C for 2 h (sampling every 0.5 h), followed by determining the residue activity. To analyze the thermostability of *Is*PETase from the high-density fermentation, the supernatant was collected and dialyzed with a Millipore 10-kDa cut-off membrane at 4 °C, followed by treated with Endo H for half an hour. Next, 0.4 mg of the enzyme was incubated in 1 mL of 50 mM glycine-NaOH buffer (pH 9.0) at 40, 45 and 50 °C for 1, 3, 6, 9, and 12 h, followed by determining the residue activity. To analyze the thermostability of Fast-PETase, the supernatant was collected and dialyzed with a Millipore 10-kDa cut-off membrane at 4 °C, followed by treated with Endo H for half an hour. Next, 0.4 mg of the enzyme was incubated in 1 mL of 100 mM KH_2_PO_4_ buffer (pH 8.0) at 50, 55, 60, 65 and 70 °C for 3, 6, 9, and 12 h, followed by determining the residual activity. Enzymes without treatment was used as the negative control. All of the experiments were carried out in triplicate.

### Kinetic analysis

To obtain kinetic parameters for MHET and BHET, a pseudo-one-substrate kinetic model was used as described previously^30^. The kinetic parameters were determined from three independent initial rate measurements performed with the same batch of purified enzymes. The protein concentration was determined using the Bradford kit (Beyotime, China). For parital**-**deglyco *Is*PETase-*Pp*, the concentration of MHET and BHET were 0.08 mM to 3 mM. The reaction was carried out at 30 °C for 2 h in 50 mM glycine-NaOH buffer (pH 9.0). For *Is*PETase-*Ec*, the concentration of MHET and BHET were 0.08 mM to 1.5 mM and 0.06 mM to 2 mM. The reaction was carried out at 30 °C in 50 mM glycine-NaOH buffer (pH 9.0) for 3 and 1 h, respectively. The initial rate was measured by measuring the released TPA using HPLC. Initial rate data were fitted to a Michaelis-Menten equation, and the *K*_M_ and *k*_*cat*_ values were calculated from the slope and y-intercept of the double-reciprocal plot.

### Depolymerization of the raw, untreated postconsumer-PET

Hole-punched postconsumer-PET flake (6 mm in diameter, approximately 7 mg) from a black and a white PET tray (Jiaduofu, China) were used as the substrate. The crystallinity of these PET trays were determined with differential scanning calorimeter (DSC). The temperature of the samples (6–9 mg) were increased from 30 to 350 °C at 5 °C/min and held at 350 °C for 1 min, followed by cooling from 350 to 30 °C at −5 °C/min and holding at 30 °C for 1 min in a DSC 200F3 Maia (NETZSCH). The percentage crystallinity was determined on the first heating scan using the enthalpies of melting and cold crystallization. The equation used to calculate percentage crystallinity within the PET film was the following:

$$\% {crystallinity}=\left[\frac{\varDelta {H}_{m}-\varDelta {H}_{{cc}}}{\varDelta H}\right]\times 100 \%$$

*ΔH*_*m*_ is the enthalpy of melting (J/g), *ΔH*_*cc*_ is the enthalpy of cold crystallization (J/g), and *ΔH* is the enthalpy of melting for a 100% crystalline PET sample, which is 140.1 J/g^10^. *ΔH*_*m*_ and *ΔH*_*cc*_ was calculated using the NETZSCH Proteus software package.

To test the activity of the partial-deglyco *Is*PETase-*Pp*, 0.8 mg of enzyme was added to a PET flake in 2 mL of 50 mM glycine-NaOH buffer (pH 9.0) and incubated at 50 °C. The supernatant was collected every 12 h to determine the released monomers and fresh enzyme solution was replenished to the vessel. To test the activity of the deglycosylated Fast*-*PETase-*Pp*, 0.8 mg of enzyme was added to a PET flake in 2 mL of 100 mM KH_2_PO_4_ buffer (pH 8.0) and incubated at 50 °C. The supernatant was collected every 12 or 24 h to determine the released monomers and fresh enzyme solution was replenished to the vessel.

To test the deploymerization of untreated PET containers with recombinant PETases, a black or a white PET tray was squeezed as a whole piece into a DURAN bottle containing 15 mg enzyme in 250 mL of 100 mM KH_2_PO_4_ buffer (pH 8.0) and incubated at 50 °C or 55 °C. Fresh enzyme solution was replenished every 24 h until most of PET was deploymerized.

### Statistics and reproducibility

Data are shown as individual data point and mean±standard deviation. All experiments presented in the manuscript were reproducible. All experiments were repeated at least twice.

### Reporting summary

Further information on research design is available in the Nature Portfolio Reporting Summary linked to this article.

## Supplementary information

Supplementary Information-New

Description of Additional Supplementary Files

supplementray data1

reporting summary

## Data Availability

Uncropped gel images are provided as Supplementary Fig. 16. Source data is provided as Supplementary Data 1. The newly generated plasmids have been deposited to the Addgene under accession numbers 195561,195562,195563 and 195565 respectively.
