# Supplementary material for: Improving the activity and thermostability of PETase from Ideonella sakaiensis through modulating its post-translational glycan modification
Source: Commun Biol. 2023 Jan 13;6:39. doi: 10.1038/s42003-023-04413-0 (PMC9839772; doi:10.1038/s42003-023-04413-0)
Supplement: Supplementary file 1 — Supplementary Information-New [file 42003_2023_4413_MOESM1_ESM.pdf]

Supplementary materials

Supplementary Table 1 Sequences of the proteins expressed in the present study

The amino acid sequence of *Is*PETase

```
        10         20         30         40         50         60
MNFPRASRLM QAAVLGGLMA VSAAATAQTN PYARGPNPTA ASLEASAGPF TVRSFTVSRP

        70         80         90        100        110        120
SGYGAGTVYY PTNAGGTVGA IAIVPGYTAR QSSIKWWGPR LASHGFVVIT IDTNSTLDQP

       130        140        150        160        170        180
SSRSSQQMAA LRQVASLNGT SSSPIYGKVD TARMGVMGWS MGGGGSLISA ANNPSLKAAA

       190        200        210        220        230        240
PQAPWDSSTN FSSVTVPTLI FACENDSIAP VNSSALPIYD SMSRNAKQFL EINGGSHSCA

       250        260        270        280        290
NSGNSNQALI GKKGVAWMKR FMDNDTRYST FACENPNSTR VSDFRTANCS
```

* The highlighted yellow region is the native signal peptide of *Is*PETase. This peptide was removed when expressed in *E. coli*. All eight potential N-linked glycosylation sites are highlighted with gray color.

The amino acid sequence of Fast-PETase

```
        10         20         30         40         50         60
MNFPRASRLM QAAVLGGLMA VSAAATAQTN PYARGPNPTA ASLEASAGPF TVRSFTVSRP

        70         80         90        100        110        120
SGYGAGTVYY PTNAGGTVGA IAIVPGYTAR QSSIKWWGPR LASHGFVVIT IDTNSTLDQP

       130        140        150        160        170        180
ESRSSQQMAA LRQVASLNGT SSSPIYGKVD TARMGVMGWS MGGGGSLISA ANNPSLKAAA

       190        200        210        220        230        240
PQAPWHSSTN FSSVTVPTLI FACENDSIAP VNSSALPIYD SMSQNAKQFL EIKGGSHSCA

       250        260        270        280        290
NSGNSNQALI GKKGVAWMKR FMDNDTRYST FACENPNSTA VSDFRTANCS
```

* The highlighted yellow region is the native signal peptide of *Is*PETase. All eight potential N-linked glycosylation sites are highlighted with gray color. All five mutation sites are highlighted with red color.

The amino acid sequence of PNGase F

```
        10         20         30         40         50         60
MRKLLIFSIS AYLMAGIVSC KGVDSATPVT EDRLALNAVN APADNTVNIK TFDKVKNAFG
```

```
       70         80         90        100        110        120
DGLSQSAEGT FTFPADVTTV KTIKMFIKNE CPNKTCDEWD RYANVYVKNK TTGEWYEIGR

       130        140        150        160        170        180
FITPYWVGTE KLPRGLEIDV TDFKSLLSGN TELKIYTETW LAKGREYSVD FDIVYGTPDY

       190        200        210        220        230        240
KYSAVVPVIQ YNKSSIDGVP YGKAHTLGLK KNIQLPTNTE KAYLRTTISG WGHAKPYDAG

       250        260        270        280        290        300
SRGCAEWCFR THTIAINNAN TFQHQLGALG CSANPINNQS PGNWAPDRAG WCPGMAVPTR

       310        320        330        340        350
IDVLNNSLTG STFSYEYKFQ SWTNNGTNGD AFYAISSFVI AKSNTPISAP VVTN
```

Supplementary Table 2 Mass spectrum to identify the main bands in the fermentation supernatant of GS115-IP-4 after treated with Endo H

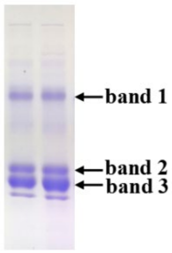

	Description	Coverage [%]	Peptides	PSMs	Unique Peptides	Sequest HT
		**band 1**				
C4R312	Dihydrolipoyl dehydrogenase	42	14	20	14	67.66
C4R3H3	SCP domain-containing protein	12	3	5	3	12.46
C4QVL4	1,3-beta-glucanosyltransferase	12	4	4	4	10.73
		**band 2**				
TARGET	TARGET	24	4	9	4	28.18
F2QQT7	Putative glucanase	5	2	8	2	21.91
F2QPL8	Endo-beta-1,3-glucanase	20	5	7	5	18.05
C4R489	Uncharacterized protein	19	4	5	4	12.45
		**band 3**				
F2QPL8	Endo-beta-1,3-glucanase	14	3	5	3	15.22
TARGET	TARGET	16	2	3	2	12.12

*TARGET indicates *Is*PETase

Supplementary Table 3 MS/MS to analyze N-linked glycosylation on *Is*PETase-*Pp*

Supplementary Table 3A Parameters for data search using PEAKS GlycanFinder software

items	value
Precursor tolerance	20.0 ppm
Fragment tolerance	0.05 Da
Modifications	Fixed：Carbamidomethylation/+57.02 Da (C)<br>Variable：Oxidation/+15.99 Da (M),<br>Deamidation/+0.98 Da (NQ)
N-linked Peptide Score	15
N-linked Glycan Score	1
Database	Sequence

Supplementary Table 3B Percentage of N-linked glycosylation for each site

site	modification type	peak Area	percentage(%)
N114	glycosylated	2.12E+04	100
	non-glycosylated	0.00E+00	0
N138	glycosylated	8.13E+05	36.57
	non-glycosylated	1.41E+06	63.43
N190	glycosylated	0.00E+00	0
	non-glycosylated	0.00E+00	0
N212	glycosylated	4.35E+04	82.23
	non-glycosylated	9.40E+03	17.77
N264	glycosylated	5.08E+05	19.78
	non-glycosylated	2.06E+06	80.22
N288	glycosylated	6.97E+04	100
	non-glycosylated	NA	0

Note: 0 of peak area indicates the concentration of the peptide is too low to gain a reading. NA indicated that no peptide is detected.

Supplementary Table 3C Component and percentage of N-linked glycan side chains for each site

N-acetylglucosamine (GlcNAc) | Galactose | Fucose

N-acetylgalactosamine (GalNAc) | Mannose | N-glycolylneuraminic acid （NeuGc）

site	component	peak area	percentage	structure
N114	(HexNAc)3(Hex)8	2.12E+04	100%	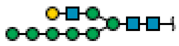
N138	(HexNAc)2(Hex)9	3.63E+05	44.64%	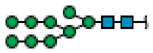
	(HexNAc)2(Hex)10	3.12E+05	38.38%	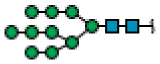

	(HexNAc)2(Hex)8	7.88E+04	9.70%	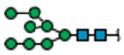
	(HexNAc)2(Hex)7	3.41E+04	4.19%	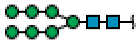
	(HexNAc)2(Hex)6	1.19E+04	1.46%	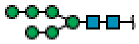
	(HexNAc)2(Hex)5	1.02E+04	1.26%	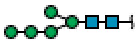
	(HexNAc)2(Hex)1	3.08E+03	0.38%	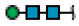
N190	(HexNAc)2(Hex)10	0	0	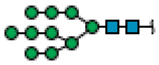
N212	(HexNAc)2(Hex)10	1.69E+04	38.83%	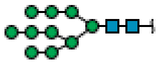
	(HexNAc)2(Hex)9	2.85E+03	31.93%	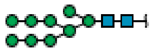
	(HexNAc)2(Hex)8	9.87E+03	22.69%	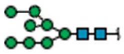
	(HexNAc)2(Hex)7	1.39E+04	6.54%	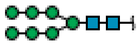
N264	(HexNAc)2(Hex)9	4.34E+05	85.38%	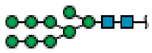
	(HexNAc)2(Hex)10	2.73E+04	5.38%	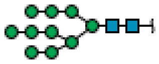
	(HexNAc)2(Hex)8	2.57E+04	5.05%	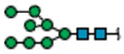
	(HexNAc)2(Hex)7	1.42E+04	2.79%	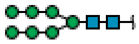
	(HexNAc)2(Hex)5	4.63E+03	0.91%	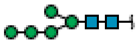
	(HexNAc)2(Hex)4	1.57E+03	0.31%	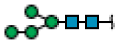
	(HexNAc)4(Hex)7	9.65E+02	0.002%	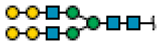
N288	(HexNAc)2(Hex)10	1.77E+04	25.44%	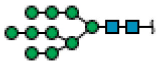
	(HexNAc)2(Hex)9	5.20E+04	74.56%	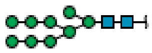

Supplementary Table 4 The main N-linked glycan side chains on Asn205 and Asn277 of *Is*PETase-*Pp* when the setting N-linked Glycan Score is 0

Supplementary Table 4A Parameters for data search using PEAKS GlycanFinder software

items	value
Precursor tolerance	20.0 ppm
Fragment tolerance	0.05 Da
Modifications	Fixed：Carbamidomethylation/+57.02 Da (C)<br>Variable：Oxidation/+15.99 Da (M)、<br>Deamidation/+0.98 Da (NQ)
N-linked Peptide Score	15
N-linked Glycan Score	0
Database	Sequence

Supplementary Table 4B Percentage of N-linked glycosylation for Asn205 and Asn277

site	modification type	area	percentage(%)
N205	glycosylated	4.19E+03	100
	non-glycosylated	0	0
N277	glycosylated	6.05E+04	100
	non-glycosylated	0	0

Note: 0 of peak area indicates the concentration of the peptide is too low to gain a reading.

Supplementary Table 4C Component and percentage of N-linked glycan side chains for Asn205 and Asn277

N-acetylglucosamine (GlcNAc) Galactose Fucose

N-acetylgalactosamine (GalNAc) Mannose N-glycolylneuraminic acid （NeuGc)

site	component	peak area	percentage	structure
N205	(HexNAc)5(Hex)3	2.23E+03	53.28%	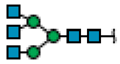
	(HexNAc)3(Hex)6(Fuc)1	1.96E+03	46.72%	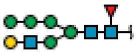
N277	(HexNAc)2(Hex)1	2.93E+04	48.43%	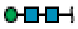
	(HexNAc)5(Hex)6(NeuGc)2	3.12E+04	51.57%	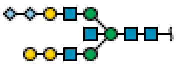

Supplementary Table 5 MS/MS to analyze O-linked glycosylation on *Is*PETase-*Pp*

Supplementary Table 5 A Parameters for data search using PEAKS GlycanFinder software

items	value
Precursor tolerance	20.0 ppm
Fragment tolerance	0.05 Da
Modifications	Fixed：Carbamidomethylation/+57.02 Da (C)<br>Variable：Oxidation/+15.99 Da (M),<br>Deamidation/+0.98 Da (NQ)
N-linked Peptide Score	15
N-linked Glycan Score	1
Database	Sequence

Supplementary Table 5 B Percentage of O-linked glycosylation for each site

site	modification type	area	percentage(%)
S7	glycosylated	5.65E+04	12.88
	non-glycosylated	3.82E+05	87.12
S22	glycosylated	5.81E+04	27.26
	non-glycosylated	1.55E+05	72.74
T26	glycosylated	1.61E+03	0.45
	non-glycosylated	3.53E+05	99.55
T29	glycosylated	7.53E+03	2.09
	non-glycosylated	3.52E+05	97.91
T39	glycosylated	7.53E+04	1.91
	non-glycosylated	3.87E+06	98.09
S42	glycosylated	4.43E+03	0.16
	non-glycosylated	2.75E+06	99.84
T51	glycosylated	1.97E+03	0.19
	non-glycosylated	1.03E+06	99.81
S58	glycosylated	8.28E+04	1.78
	non-glycosylated	4.57E+06	98.22
T67	glycosylated	2.70E+05	4.78
	non-glycosylated	5.38E+06	95.22
T72	glycosylated	1.25E+03	0.02
	non-glycosylated	6.45E+06	99.98
T77	glycosylated	6.85E+03	0.01
	non-glycosylated	6.72E+06	99.90
T151	glycosylated	6.88E+02	0.04
	non-glycosylated	1.64E+06	99.96
S166	glycosylated	1.56E+06	13.15
	non-glycosylated	1.03E+07	86.85
S169	glycosylated	1.83E+06	15.09
	non-glycosylated	1.03E+07	84.91
S175	glycosylated	3.01E+05	3.11

	non-glycosylated	9.37E+06	96.89
S242	glycosylated	3.23E+04	0.34
	non-glycosylated	9.56E+06	99.66
S245	glycosylated	3.29E+04	0.35
	non-glycosylated	9.35E+06	99.65
S266	glycosylated	1.44E+04	57.83
	non-glycosylated	1.05E+04	42.17
S269	glycosylated	1.33E+04	55.88
	non-glycosylated	1.05E+04	44.12

Supplementary Table 5C Component and percentage of O-linked glycan side chains for each site

N-acetylglucosamine (GlcNAc)　Galactose　Fucose

N-acetylgalactosamine (GalNAc)　Mannose　N-glycolylneuraminic acid （NeuAc）

N-glycolylneuraminic acid （NeuGc）　Glucose

site	component	peak area	percentage	structure
S7	(HexNAc)1	5.65E+04	100%	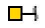
S22	(HexNAc)3	5.65E+04	97.18%	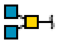
	(HexNAc)1	1.34E+03	2.30%	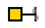
	(Hex)1	2.98E+02	0.51%	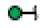
T26	(HexNAc)3	8.83E+02	54.88%	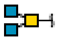
	(Hex)1	7.26E+02	45.12%	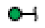
T29	(Hex)1	5.04E+03	66.95%	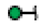
	(HexNAc)2(Hex)4(Fuc)1	2.49E+03	33.05%	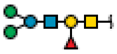
T39	(HexNAc)1(Hex)1(NeuAc)1	7.53E+04	100%	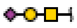
S42	(HexNAc)1(Hex)1	4.43E+03	100%	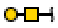
T51	(Hex)1	1.97E+03	100%	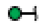
S58	(Fuc)1	8.28E+04	100%	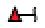
T67	(Hex)1	2.70E+05	100%	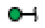
T72	(HexNAc)1(Fuc)1	1.25E+03	100%	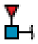
	(Fuc)1	0	0	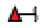
T77	(HexNAc)1(Hex)2(Fuc)2	4.64E+03	67.74%	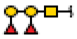

	(HexNAc)1(Fuc)1	2.21E+03	32.26%	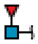
T151	(Hex)1	6.88E+02	100%	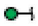
S166	(Hex)1	1.56E+06	99.57%	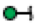
	(HexNAc)1(NeuAc)1	2.95E+03	0.43%	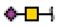
S169	(HexNAc)1(Hex)2	1.56E+06	85.31%	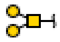
	(HexNAc)3	2.43E+05	13.32%	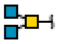
	(HexNAc)1(Fuc)1	2.08E+04	1.14%	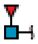
	(HexNAc)1(Hex)1(NeuAc)1	2.95E+03	0.16%	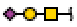
	(HexNAc)2(Hex)3(Fuc)1	9.75E+02	0.05%	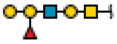
	(HexNAc)1(Hex)1(Fuc)1	3.20E+02	0.02%	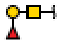
S175	(HexNAc)2(Hex)1(Fuc)1	2.58E+05	85.77%	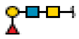
	(HexNAc)3(Hex)2(Fuc)1	3.92E+04	13.03%	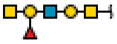
	(HexNAc)3(Hex)1(Fuc)1	2.16E+03	0.72%	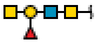
	(HexNAc)1(Hex)1(NeuAc)4	5.14E+02	0.17%	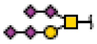
	(Hex)1(Fuc)1	3.63E+02	0.12%	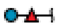
	(HexNAc)1(Hex)1(Fuc)1	3.20E+02	0.11%	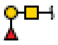
	(HexNAc)1(Hex)1(NeuAc)3	1.41E+02	0.05%	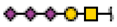
	(HexNAc)2(Hex)1(Fuc)1(NeuAc)2	9.54E+01	0.03%	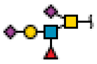
S242	(Hex)1(Fuc)1	2.73E+04	84.62%	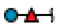
	(Fuc)1	4.96E+03	15.38%	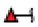
S245	(HexNAc)1(Hex)2(Fuc)2	2.73E+04	83.04%	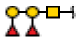
	(Hex)1(NeuAc)1	3.16E+03	9.62%	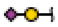
	(Fuc)1	1.81E+03	5.50%	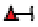
	(HexNAc)3(Hex)1(Fuc)1	5.14E+02	1.57%	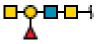

	(HexNAc)2	8.90E+01	0.27%	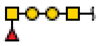
T266	(Hex)1	8.75E+03	60.84%	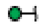
	(Fuc)1	5.63E+03	39.16%	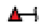
S269	(Hex)1	7.62E+03	57.49%	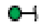
	(Hex)1(NeuAc)1	5.63E+03	42.51%	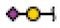

Note: 0 of peak area indicates the concentration of the peptide is too low to gain a reading.

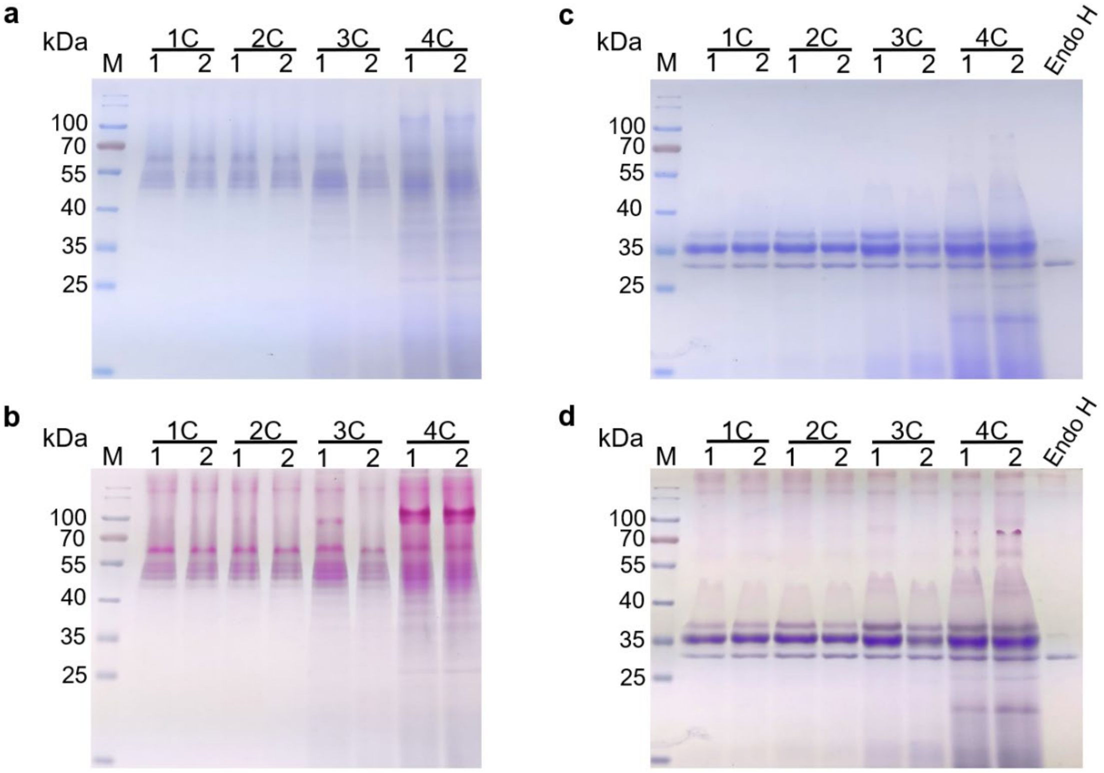

Supplementary Fig.1: The expression and deglycosylation of *Is*PETase-*Pp* prepared with the shake-flask fermentation. **a** SDS-PAGE to analyze *Is*PETase-*Pp* expressed with *P. pastoris* bearing 1-4 copies of the target gene; Two parallel samples (1, 2) were tested for each copy number. **b** Glycoprotein staining of the gel in **(a)**. **c** SDS-PAGE to analyze the same samples as **(a)** after treated with Endo H. **d** Glycoprotein staining of the gel in **(c)**. M: protein molecular weight standards (the size of each band is indicated on the left).

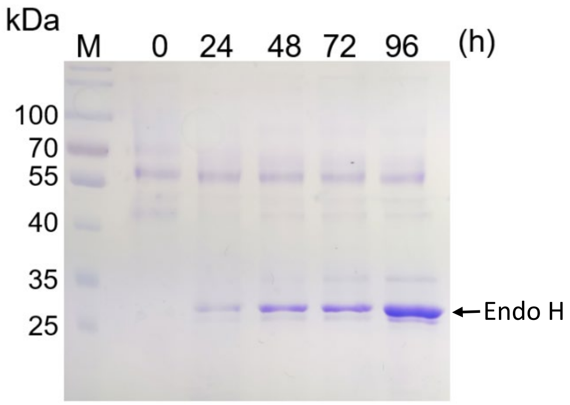

Supplementary Fig.2: The recombinant Endo H expressed with *P. pastoris* GS115. The target protein was prepared with the shake-flask fermentation, followed by centrifugation at 5000 rpm for 10 min. The supernatant was gathered and stored at 4 °C for further use. M: protein molecular weight standards (the size of each band is indicated on the left).

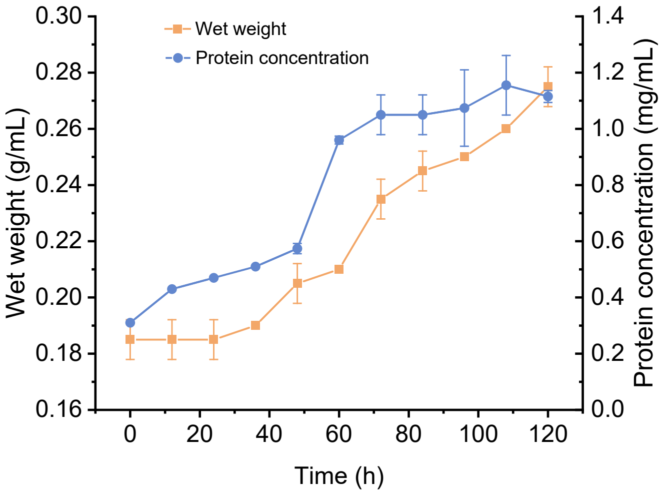

Supplementary Fig.3: The time course of the total enzyme activity and protein concentration in the supernatant of the cell culture during the inducing phase of the high-density fermentation. Data are presented as mean ± SD.

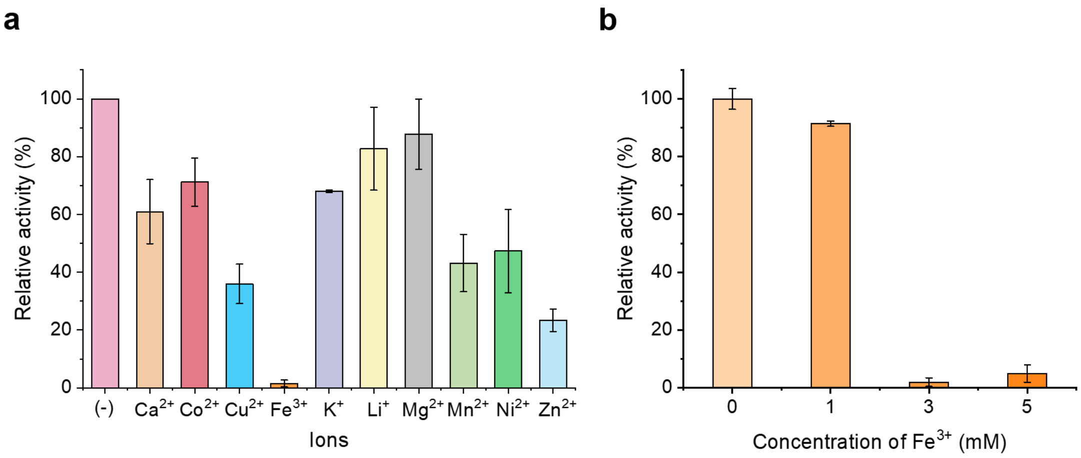

Supplementary Fig.4: Effect of metal cations on the activity of *Is*PETase-*Pp*. a The catalytic activities of *Is*PETase-*Pp* in the presence of various metal ions (3 mM). The assay was carried out by adding 200 μL of the diluted enzyme (0.4 mg) to 3 mg of amorphous PET in 50 mM glycine-NaOH buffer (pH 9.0) supplemented with 3 mM of different ions to a final volume of 1 mL, followed by incubating at 30 °C for 6 h. The reaction was terminated by heating at 85 °C for 10 min, and the mixture was centrifuged at 14000 g for 1 min, followed by analysis with HPLC. The activity of the enzyme without extra ions was set to 100%. **b** The catalytic activities of *Is*PETase-*Pp* in the presence of ferric ion of 0, 1, 3 and 5 mM, respectively. Data are presented as mean ± SD.

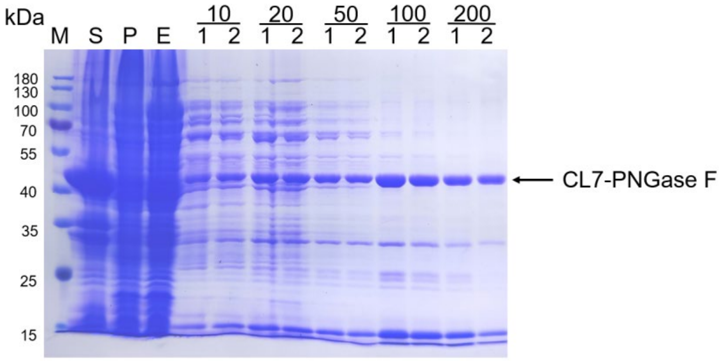

Supplementary Fig.5: Expression of CL7-PNGase F with *E. coli* and purification with affinity chromatography. S: supernatant of the cell lysate; P: pellet of the cell lysate; E: flow-through; 10, 20, 50, 100, 200 indicates the concentrations of imidazole (mM) used for elution. Two parallel samples (1, 2) were loaded for each concentration. M: protein molecular weight standards (the size of each band is indicated on the left).

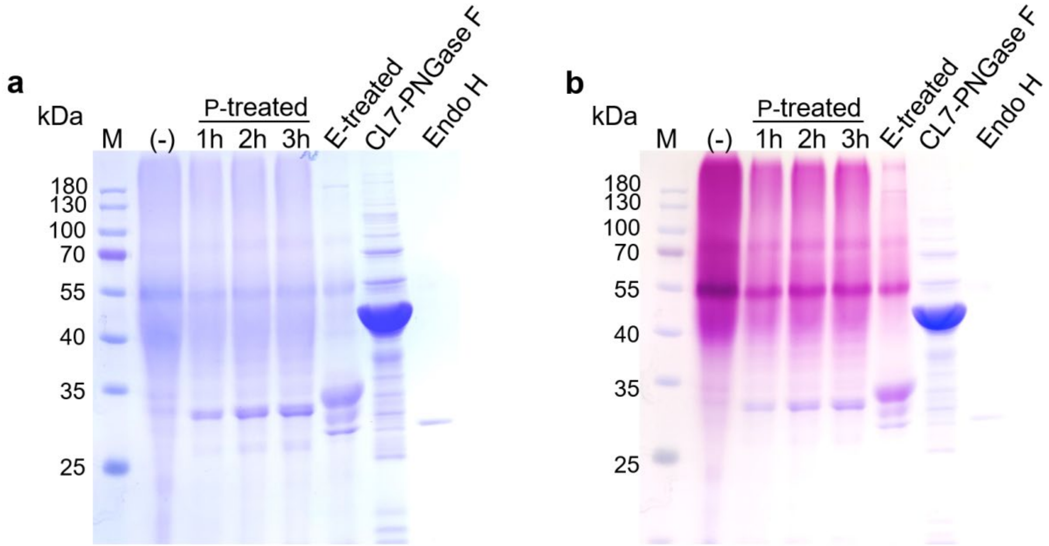

Supplementary Fig.6: The treatment of *Is*PETase-*Pp* with CL7-PNGase F. a SDS-PAGE to analyze *Is*PETase-*Pp* treated with CL7-PNGase F; b Glycoprotein staining of the gel from **(a).** (-): *Is*PETase-*Pp* without treatment. P-treated: *Is*PETase-*Pp* digested with CL7-PNGase F for 1, 2, and 3 h, respectively; E-treated: *Is*PETase-*Pp* digested with Endo H for half an hour; M: protein molecular weight standards (the size of each band is indicated on the left).

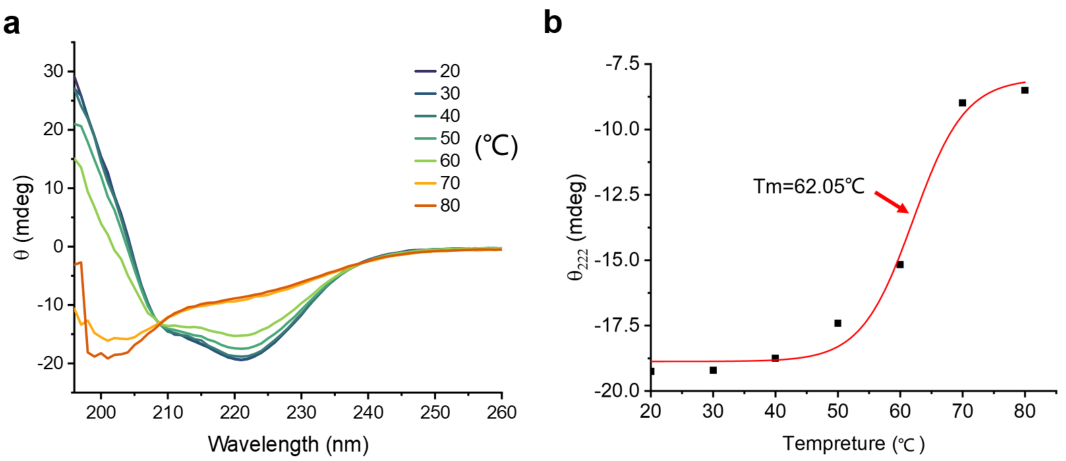

Supplementary Fig.7: Far-ultraviolet CD to determine the T_m of partial-deglyco *Is*PETase-*Pp*. **a** Far-ultraviolet CD graphs of partial-deglyco *Is*PETase-*Pp* from 20 to 80 °C. **b** Melting curve of parital-deglyco *Is*PETase-*Pp*.

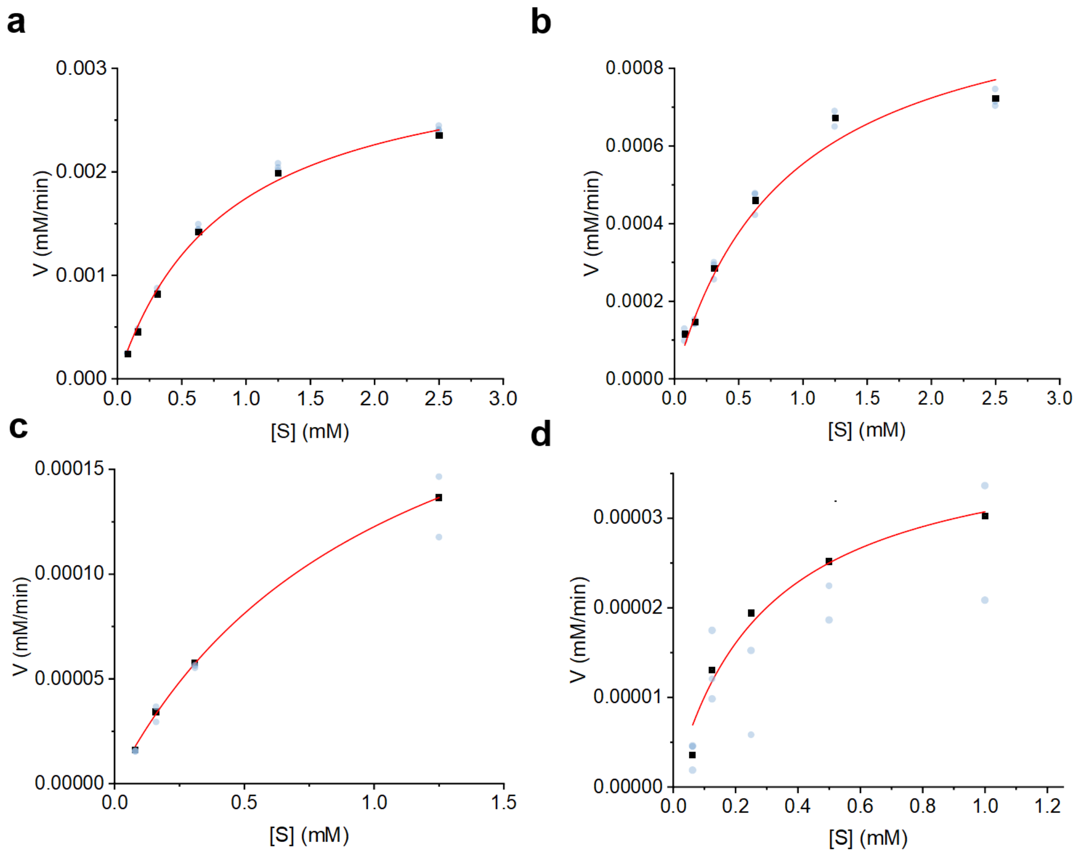

Supplementary Fig.8: Kinetics curves for enzymatic activities of partial-deglyco *Is*PETase-*Pp* and *Is*PETase-*Ec* with MHET and BHET as substrates. a parital-deglyco *Is*PETase-*Pp* with MHET as the substrate. **b** parital-deglyco *Is*PETase-*Pp* with BHET as the substrate. **c** *Is*PETase-*Ec* with MHET as the substrate. **d** *Is*PETase-*Ec* with BHET as the substrate. For parital-deglyco *Is*PETase-*Pp*, the concentration of MHET and BHET were 0.08 mM to 3 mM. The reaction was carried out at 30 °C for 2 h in 50 mM glycine-NaOH buffer (pH 9.0) . For *Is*PETase-*Ec*, the concentration of MHET and BHET were 0.08 mM to 1.5 mM and 0.06 mM to 2 mM. The reaction was carried out at 30 °C in 50 mM glycine-NaOH buffer (pH 9.0) for 3 and 1 h, respectively. The initial rate was measured by measuring the released TPA using HPLC.

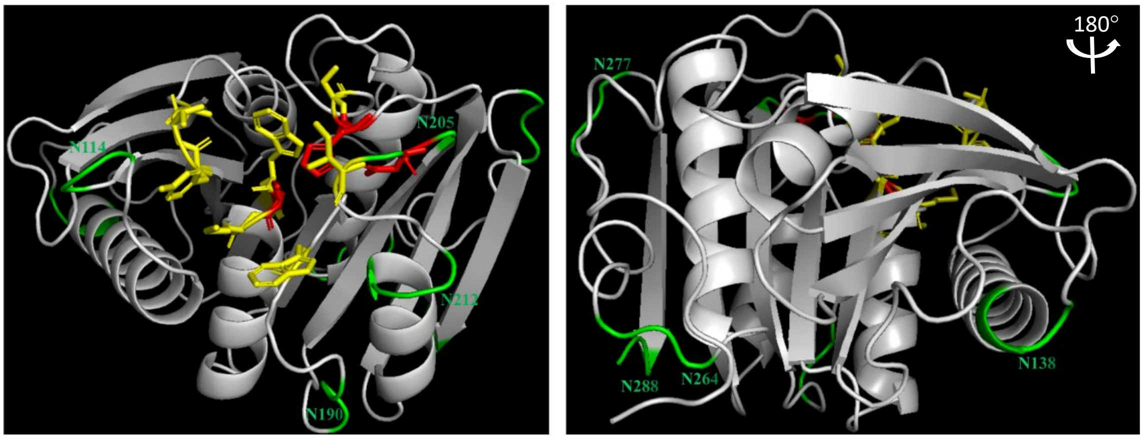

Supplementary Fig.9: The potential glycosylation sites in *Is*PETase (PDB: 6ANE). The eight potential N-linked glycosylation sites are labeled in green. The traid and the key amino acid residues involved in catalysis are labeled in red and yellow, respectively.

N205

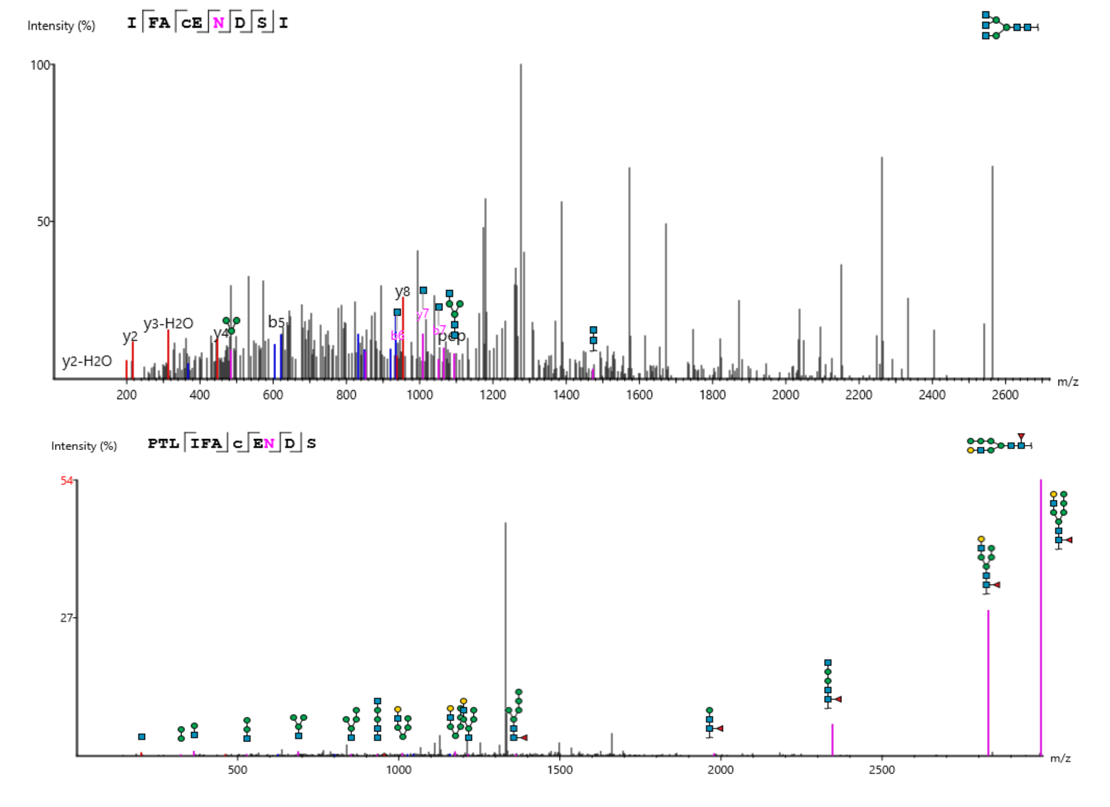

Intensity (%)
I FA cE N D S I
100
50
y2-H2O
y2
y3-H2O
y4
b5
y8
y7
b6
b7
pep
m/z
Intensity (%)
PTL IFA c EN D S
54
27
m/z

N277

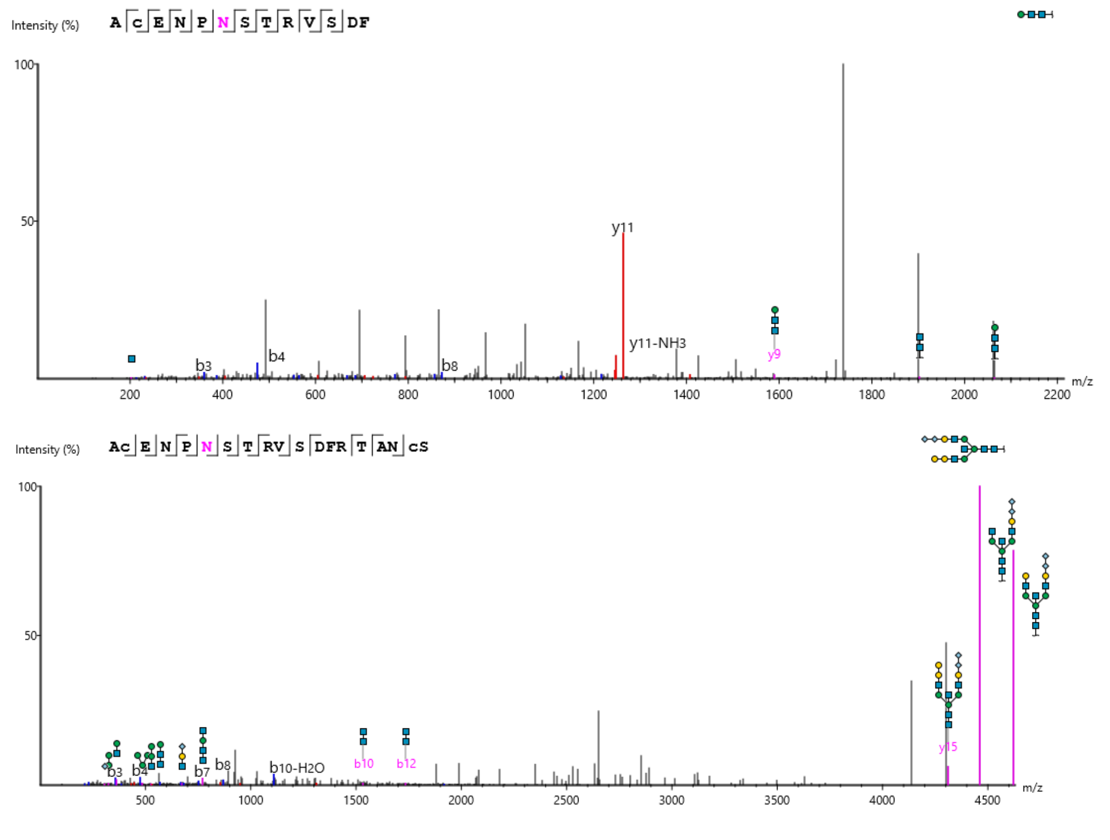

Intensity (%)
A c E N P N S T R V S DF
100
50
y11
y11-NH3
b3
b4
b8
y9
m/z
Intensity (%)
Ac E N P N S T RV S DFR T AN cS
100
50
b3
b4
b7
b8
b10-H2O
b10
b12
y15
m/z

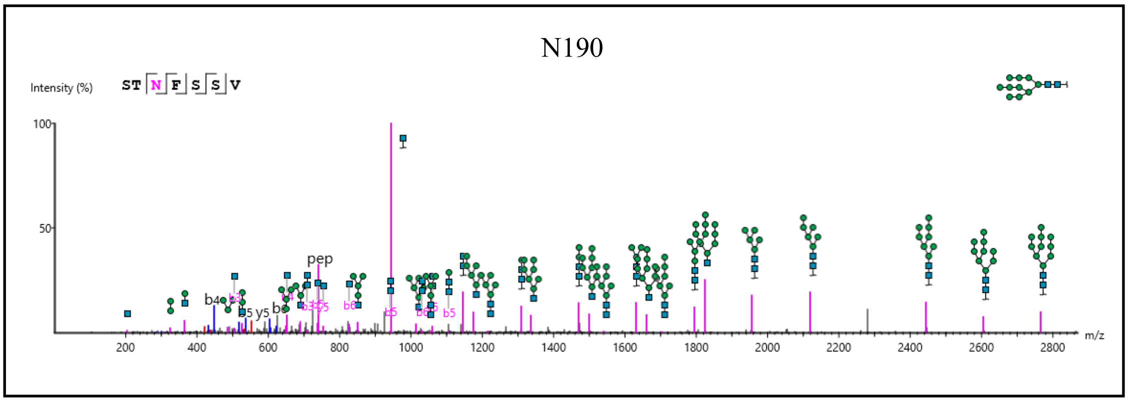

Supplementary Fig.10: The MS/MS maps of the main N-linked glycan side chains on Asn205, Asn277 and Asn190.

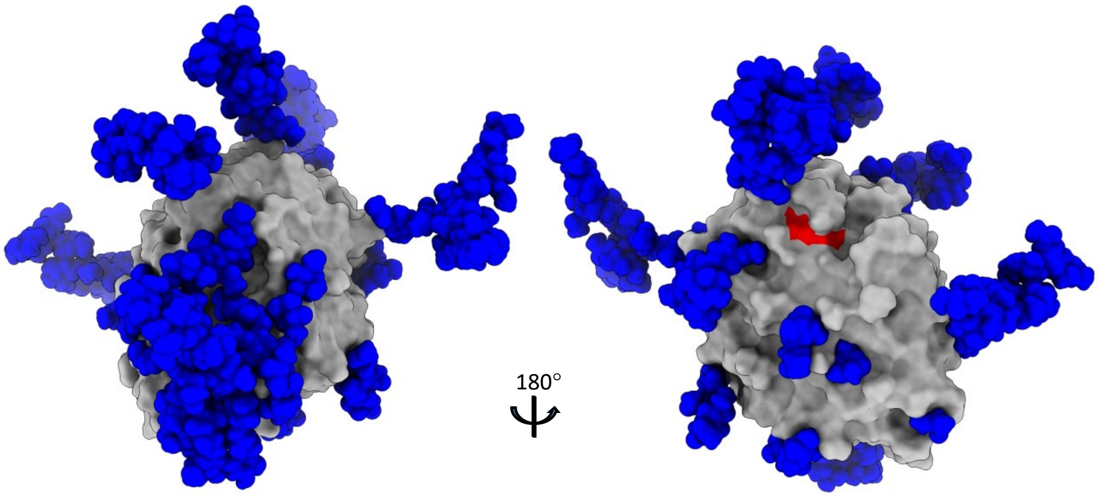

Supplementary Fig.11: The structure modeling of glycosylated *Is*PETase-*Pp* (PDB: 6ANE). The glycoprotein was build at GLYCAM-Web (www.glycam.org) and the figure was illustrated with UCSF chimera. The protein is indicated in gray while N- and O-glycan side chains are indicated in blue. The catalytic center is indicated in red.

Note: According to the result of MS/MS, several glycosylation sites are modified with various glycan types. To simplify the model, the dominant glycan types (with the highest peak area) are chosen to construct the three-dimensional model.

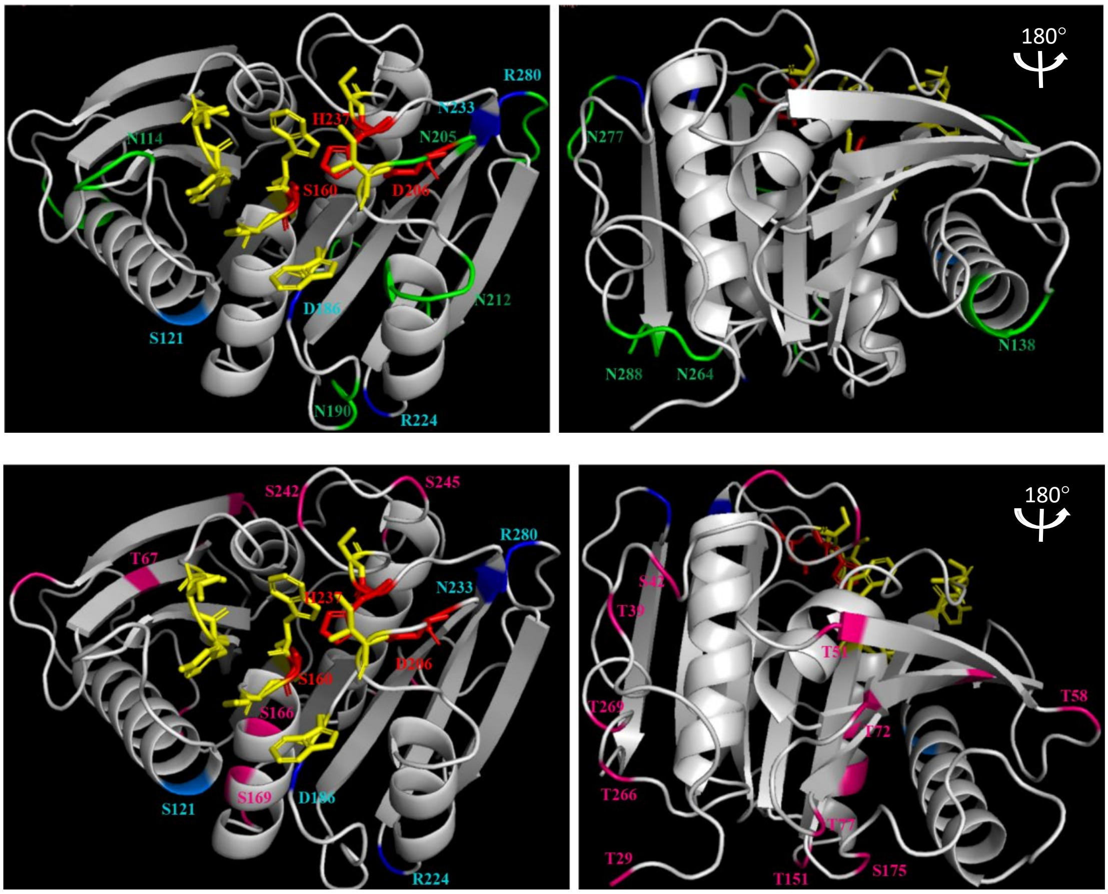

Supplementary Fig.12: The mutation sites in Fast-PETase and the residues occupied by N-linked and O-linked glycosylation on *Is*PETase-*Pp* (PDB: 6ANE). The traid of *Is*PETase are labeled in red. The key residues involved in catalysis are labeled in yellow. The mutation sites in Fast-PETase are labeled in blue. The residues occupied by N-linked and O-linked glycosylation are labeled in green and hotpink, respectively.

Note: S7, S22 and T26 are not shown in the Figure because the first 28 amino acids are not included in the structure.

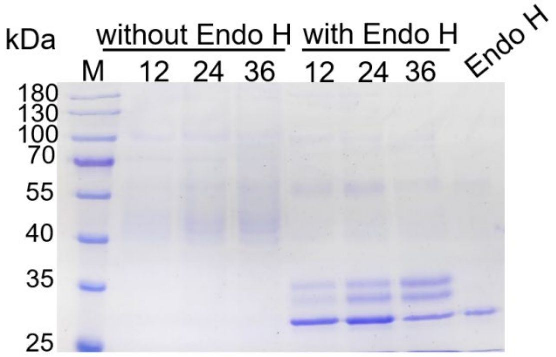

Supplementary Fig.13: Expression of Fast-PETase-*Pp* with the shake-flask fermentation. The supernatant of the cell culture was collected every 12 h and treated with Endo H for half an hour at 30 °C.

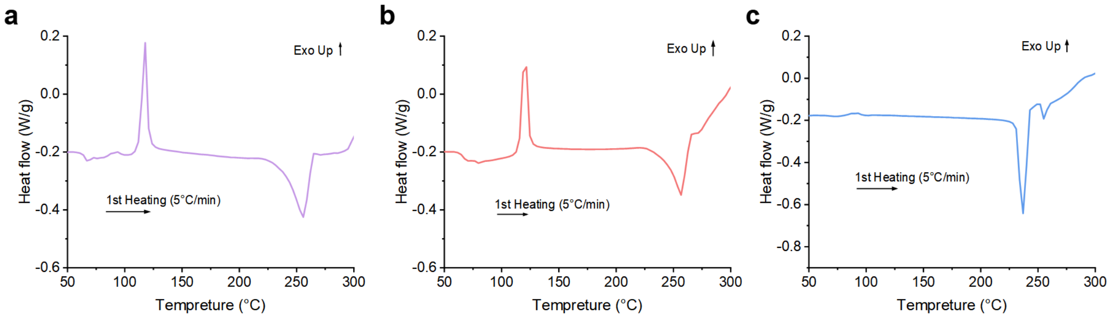

Supplementary Fig.14: DSC to determine the percentage crystallinity of postconsumer-PET. a The percentage crystallinity of the black tray. The melting onset is 252.9 °C. The melting peak temperature is 256.6 °C. The glass transition temperature is 62.3 °C. **b** The percentage crystallinity of the white tray. The melting onset is 242.0 °C. The melting peak temperature is 257.9 °C. The glass transition temperature is 66.2 °C. **c** The percentage crystallinity of amorphous PET. The melting onset is 231.9 °C. The melting peak temperature is 237.2 °C. The glass transition temperature is 70.5 °C.

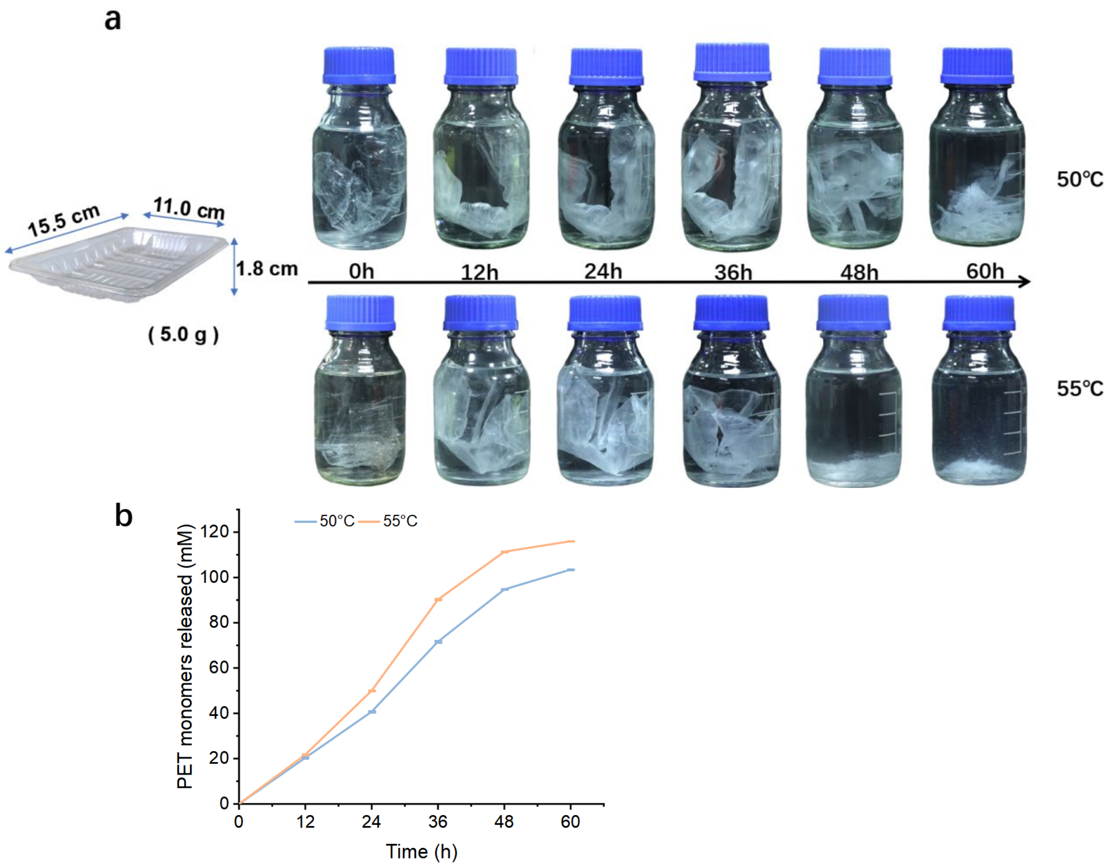

Supplementary Fig.15: Depolymerization of the raw, untreated postconsumer-PET with partial-deglyco FAST-PETase-*Pp*. **a** Degradation of untreated PET trays with partial-deglyco FAST-PETase-*Pp* at 50 °C and 55 °C, respectively. **b** Time course of PET monomers released from the flakes during the digestion. Data are presented as mean ± SD.

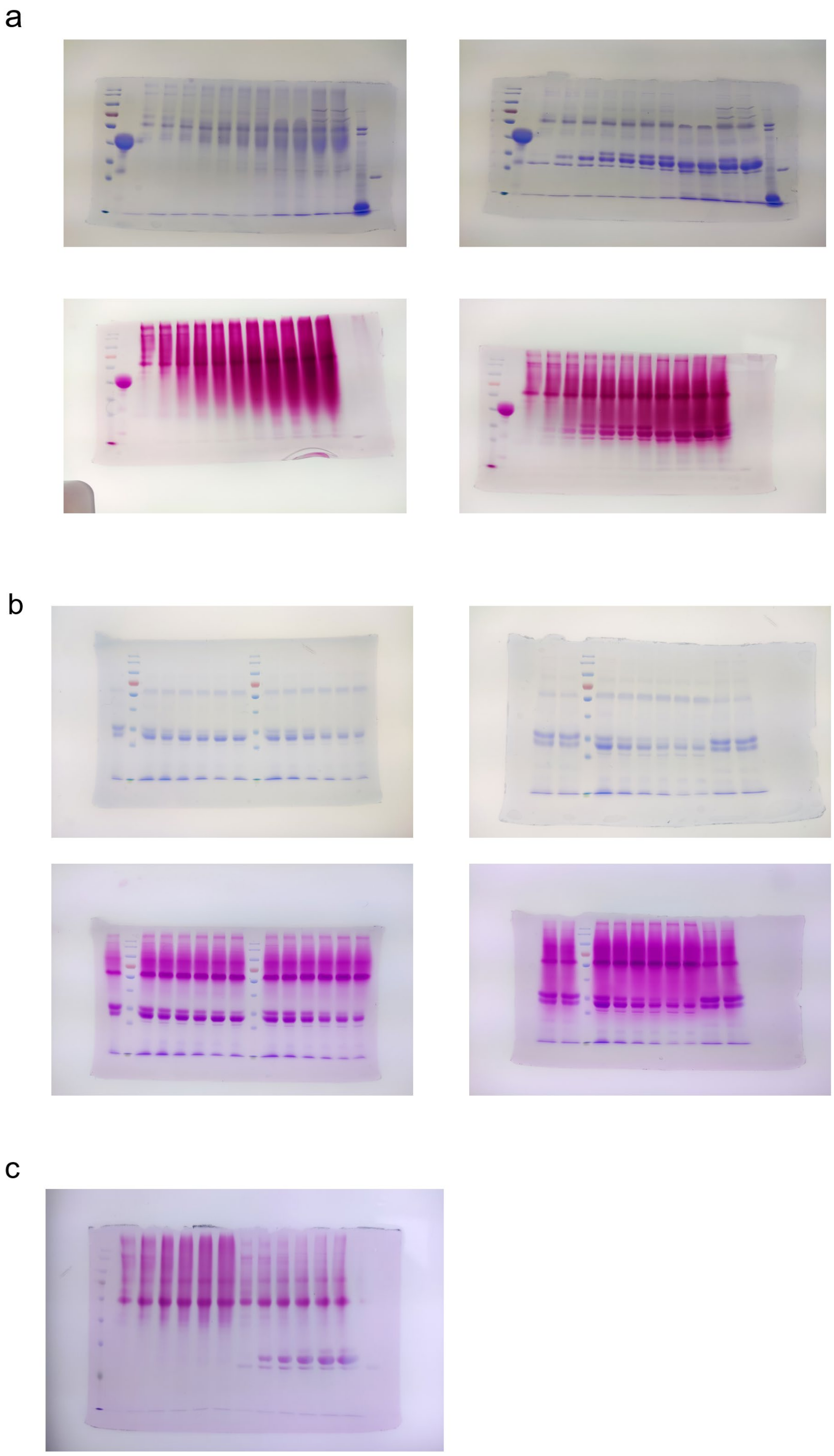

Supplementary Fig.16: Uncropped and unedited SDS-PAGE gels. a The uncropped SDS-PAGE gel of Fig.2. **b** The uncropped SDS-PAGE gel of Fig.4. **c** The uncropped SDS-PAGE gel of Fig.5.
