# Supplementary material for: Improving the activity and thermostability of PETase from Ideonella sakaiensis through modulating its post-translational glycan modification
Source: Commun Biol. 2023 Jan 13;6:39. doi: 10.1038/s42003-023-04413-0 (PMC9839772; doi:10.1038/s42003-023-04413-0)
Supplement: Supplementary file 2 — Description of Additional Supplementary Files [file 42003_2023_4413_MOESM2_ESM.docx]

**Description of Additional Supplementary Files**

File name: Supplementary Data 1

Description: The data used for ploting graphs in Fig 1 to Fig 6.
