# Supplementary material for: Improving the activity and thermostability of PETase from Ideonella sakaiensis through modulating its post-translational glycan modification
Source: Commun Biol. 2023 Jan 13;6:39. doi: 10.1038/s42003-023-04413-0 (PMC9839772; doi:10.1038/s42003-023-04413-0)
Supplement: Supplementary file 4 — reporting summary [file 42003_2023_4413_MOESM4_ESM.pdf]

nature portfolio

Corresponding author(s): Chao Zhai, Lixin Ma

Last updated by author(s): Nov 30, 2022

Reporting Summary

Nature Portfolio wishes to improve the reproducibility of the work that we publish. This form provides structure for consistency and transparency in reporting. For further information on Nature Portfolio policies, see our Editorial Policies and the Editorial Policy Checklist.

Statistics

For all statistical analyses, confirm that the following items are present in the figure legend, table legend, main text, or Methods section.

n/a	Confirmed	
☐	☒	The exact sample size (*n*) for each experimental group/condition, given as a discrete number and unit of measurement
☐	☒	A statement on whether measurements were taken from distinct samples or whether the same sample was measured repeatedly
☐	☒	The statistical test(s) used AND whether they are one- or two-sided<br>*Only common tests should be described solely by name; describe more complex techniques in the Methods section.*
☐	☒	A description of all covariates tested
☐	☒	A description of any assumptions or corrections, such as tests of normality and adjustment for multiple comparisons
☐	☒	A full description of the statistical parameters including central tendency (e.g. means) or other basic estimates (e.g. regression coefficient) AND variation (e.g. standard deviation) or associated estimates of uncertainty (e.g. confidence intervals)
☒	☐	For null hypothesis testing, the test statistic (e.g. *F*, *t*, *r*) with confidence intervals, effect sizes, degrees of freedom and *P* value noted<br>*Give P values as exact values whenever suitable.*
☒	☐	For Bayesian analysis, information on the choice of priors and Markov chain Monte Carlo settings
☒	☐	For hierarchical and complex designs, identification of the appropriate level for tests and full reporting of outcomes
☒	☐	Estimates of effect sizes (e.g. Cohen's *d*, Pearson's *r*), indicating how they were calculated

Our web collection on statistics for biologists contains articles on many of the points above.

Software and code

Policy information about availability of computer code

Data collection	pymol for the structure of PETase;<br>Q ExactiveTM HF mass spectrometry to identify proteins;<br>tims TOF Pro2 mass spectrum to analyze glycoproteins;<br>Chirascan V100 circle dichroism spectrometer to analyze Tm of PETase.
Data analysis	Proteome Discoverer 2.4 software to identify proteins;<br>PEAKS GlycanFinder software to analyze glycoproteins;<br>OriginPro 8.0 software to calculate kinetic parameters of PETase;<br>UCSF chimera software to illustrate 3D structure of the PETase glycoprotein.

For manuscripts utilizing custom algorithms or software that are central to the research but not yet described in published literature, software must be made available to editors and reviewers. We strongly encourage code deposition in a community repository (e.g. GitHub). See the Nature Portfolio guidelines for submitting code & software for further information.

Data

Policy information about availability of data

All manuscripts must include a data availability statement. This statement should provide the following information, where applicable:

- Accession codes, unique identifiers, or web links for publicly available datasets
- A description of any restrictions on data availability
- For clinical datasets or third party data, please ensure that the statement adheres to our policy

Data that support the findings of this study are available from the corresponding authors upon request. The structure of PETase used in this paper were taken from PDB (accession codes specified in the figure captions).The source data underlying figures 1-6 , Table 1-2, supplementary Tables 1-3, and supplementary figures 1-12 are provided as Source Data files. GLYCAM-Web (www.glycam.org) was applied to construct 3D structure of the PETase glycoprotein.

Human research participants

Policy information about studies involving human research participants and Sex and Gender in Research.

Reporting on sex and gender	N/A
Population characteristics	N/A
Recruitment	N/A
Ethics oversight	N/A

Note that full information on the approval of the study protocol must also be provided in the manuscript.

Field-specific reporting

Please select the one below that is the best fit for your research. If you are not sure, read the appropriate sections before making your selection.

☒ Life sciences ☐ Behavioural & social sciences ☐ Ecological, evolutionary & environmental sciences

For a reference copy of the document with all sections, see nature.com/documents/nr-reporting-summary-flat.pdf

Life sciences study design

All studies must disclose on these points even when the disclosure is negative.

Sample size	Sample size was chosen based on previous studies (Son, H. F. et al. Rational Protein Engineering of Thermo-Stable PETase from Ideonella sakaiensis for Highly Efficient PET Degradation. ACS Catalysis (2019);Lu, H. et al. Machine learning-aided engineering of hydrolases for PET depolymerization. Nature 604, 662-667, (2022)) . No statistical methods were used to predetermine the sample size.
Data exclusions	To identify proteins with MS (supplementary Table 2), proteins with Sequest HT<10 were eliminated. To identify the glycan side chains of PETase, the signals with peak areas of less than E+03 were eliminated.
Replication	All of the experiments were carried out in triplicate.
Randomization	The PET trays were purchased randomly on-line. To study the expression level of the recombinant strains, 20 transformants were picked randomly after the electroporation and identified with whole-cell PCR. Next, three positive colonies were picked for the shake-flask fermentation. And the colony with the highest expression yield was chosen for the high-density fermentation.
Blinding	Blinding is not necessary in this study because the experiments are based on preexisting data from other sources, the analysis is down by software without human intervention and the results are not expected to be influenced by subjective factors.

Reporting for specific materials, systems and methods

We require information from authors about some types of materials, experimental systems and methods used in many studies. Here, indicate whether each material, system or method listed is relevant to your study. If you are not sure if a list item applies to your research, read the appropriate section before selecting a response.

Materials & experimental systems

n/a	Involved in the study
☒	☐ Antibodies
☒	☐ Eukaryotic cell lines
☒	☐ Palaeontology and archaeology
☒	☐ Animals and other organisms
☒	☐ Clinical data
☒	☐ Dual use research of concern

Methods

n/a	Involved in the study
☒	☐ ChIP-seq
☒	☐ Flow cytometry
☒	☐ MRI-based neuroimaging
